# Estimating the impact of donor programs on child mortality in low- and middle-income countries: a synthetic control analysis of child health programs funded by the United States Agency for International Development

**DOI:** 10.1186/s12963-021-00278-9

**Published:** 2022-01-06

**Authors:** William Weiss, Bhumika Piya, Althea Andrus, Karar Zunaid Ahsan, Robert Cohen

**Affiliations:** 1 Department of International Health, John Hopkins University & Public Health Institute (USAID Contractor), 615 N. Wolfe Street, Rm E8132, Baltimore, MD 21205 USA; 2 Global Programs, Water For People, 100 E. Tennessee Ave, Denver, CO 80209 USA; 3 Alutiiq (State Department Contractor), 2000 N. Adams St., Arlington, VA 22201 USA; 4 UNC Center for Health Equity Research, School of Medicine, The University of North Carolina at Chapel Hill, 323 MacNider Hall 333 South Columbia Street, Chapel Hill, NC 27599-7240 USA; 5 Camris International (USAID Contractor), 3 Bethesda Metro Center, 16th Floor, Bethesda, MD 20814 USA

**Keywords:** Synthetic control analysis, Integrated Management of Childhood Illness (IMCI), Low- and middle-income countries (LMICs), United States Agency for International Development (USAID), Child mortality, Quasi-experimental methods, Maternal and child health (MCH), Impact evaluation, Donor assistance

## Abstract

**Background:**

Significant levels of funding have been provided to low- and middle-income countries for development assistance for health, with most funds coming through direct bilateral investment led by the USA and the UK. Direct attribution of impact to large-scale programs funded by donors remains elusive due the difficulty of knowing what would have happened without those programs, and the lack of detailed contextual information to support causal interpretation of changes.

**Methods:**

This study uses the synthetic control analysis method to estimate the impact of one donor’s funding (United States Agency for International Development, USAID) on under-five mortality across several low- and middle-income countries that received above average levels of USAID funding for maternal and child health programs between 2000 and 2016.

**Results:**

In the study period (2000–16), countries with above average USAID funding had an under-five mortality rate lower than the synthetic control by an average of 29 deaths per 1000 live births (year-to-year range of − 2 to − 38). This finding was consistent with several sensitivity analyses.

**Conclusions:**

The synthetic control method is a valuable addition to the range of approaches for quantifying the impact of large-scale health programs in low- and middle-income countries. The findings suggest that adequately funded donor programs (in this case USAID) help countries to reduce child mortality to significantly lower rates than would have occurred without those investments.

**Supplementary Information:**

The online version contains supplementary material available at 10.1186/s12963-021-00278-9.

## Background

Substantial resources have been provided to low- and middle-income countries (LMICs) to support the development of the health sector. Close to $600 billion of development assistance for health (DAH) was provided between 1995 and 2018 [1]. In 2018 alone, the DAH was estimated at $38.9 billion with the majority of funds coming through direct bilateral assistance, with the USA leading at 34% of total DAH followed by the UK at 8.4%. Of the total 2018 DAH, 24% ($9.5 billion) went to HIV/AIDS, 20.1% ($7.8 billion) went to newborn and child health, 14.3% ($5.6 billion) went to health systems strengthening, and 12.1% ($4.7 billion) went to reproductive and maternal health [1]. Multilateral development agencies and other private–public partnerships, such as the Global Fund, The World Health Organization (WHO), World Bank, the Bill and Melinda Gates Foundation, and the United Nations Children's Fund (UNICEF) jointly disbursed approximately 31% of total DAH in 2018 [2]. Single-year estimates of DAH specifically for reproductive, maternal and child health vary by source with estimates ranging from $10.8 to $13.1 billion, with the USA consistently as the single largest contributor across sources [3].

Given this large investment, donors of health programs want evidence that health has improved in the communities served by these donors’ programs. This is desired in order to be accountable for the funding that donor agencies receive, as well as to justify continued funding for these programs [4]. For example, the Bill and Melinda Gates Foundation (BMGF) has a website for press releases that allow the Foundation to publicize its maternal and child health programs [5]. The United Kingdom’s Foreign, Commonwealth & Development Office (FCDO) has a Development Tracker where one can find reports on the results of the health programs it funds [6]. The United States Agency for International Development (USAID) has a document library called the Development Experience Clearinghouse with annual reports to Congress that have information about programs aiming to prevent child deaths and their results [7].

In addition, to make an even stronger case to constituencies that a donor’s program is valuable, it is desirable to have evidence that can directly attribute the cause of positive changes in patient or population health to that specific donor’s program. Evidence of such attribution is rare, however, as it requires a difficult-to-achieve evaluation design that justifies causal inference about the effects of the program [8]. Evidence of attribution is particularly difficult in low- and middle-income countries with a history of donor support [9]. As Victora et al. articulate very well, traditional evaluation designs that compare a donor-funded intervention in a program area with no intervention in a control area may no longer be feasible. Health programs, including child health programs relevant to this study, have been and are being scaled up nationwide, and the existence of potential comparison areas or populations—that have not experienced donor-supported child health interventions—is rare. Thus, traditional evaluation designs may not provide a credible counterfactual necessary for causal inference. And, it is hard to tease out benefits of a program from one donor when there are many donor programs in a country and most/all work in partnership with host government and communities, without detailed information about these programs and the contextual factors that would allow for plausible interpretations of impact [10, 11].

Victora et al. argue for the development of a national evaluation platform in each country with the district as the main unit of analysis that *‘includes documentation of contextual factors and implementation of many programs—and indicators of coverage, impact, and cost”* (pg 94) [9]. In each country, data would be compiled from existing population-based sources and from routinely collected data from health facilities and administrative sources. These data would reflect a conceptual model around maternal and child health interventions leading to reduced mortality [12]. The quality of data would be assessed and improved. Data would then be organized to facilitate cross-district analysis by time and equity. This approach may be considered an optimal approach given the difficulties with evaluation in LMICs using traditional methods. However, this approach might be considered a long-term solution as platforms integrating population information (vital registration, household surveys) with facility-based and administrative statistics at the district-level on a routine basis do not currently exist in most LMICs. Developing such platforms would require considerable expertise and resources [13].

This paper provides an alternative, interim method to estimate the impact of donor funding on health outcomes, in the absence of detailed contextual information. Using the case of a single donor’s effort (USAID), we use a synthetic cohort analysis methodology to quantify the impact of USAID funding on under-five mortality across several low- and middle-income countries. The analysis presented here provides a data-driven strategy to produce a counterfactual (i.e., a circumstance with no intervention) to quantify the impact. The synthetic control method has been used in high-, middle- and low-income countries [14–17]. Abadie, Diamond, and Hainmueller describe the utility of the synthetic control method (Additional file 1: Section S1) [14].

In this study, we apply a novel method (synthetic control) to estimate the impact of donor investments in child health. Specifically, we attempt to quantify the impact of USAID investment and support on child mortality. We test the method by observing a particular scenario where we believe the impact of USAID investments can be quantified using a synthetic control approach. The scenario takes place in countries with relatively high levels of USAID support for child health during the period of the World Health Organization’s initiative to accelerate reductions in child mortality, called the Integrated Management of Childhood Illness (IMCI) and Integrated Community Case Management (iCCM) (1999–2016).

### Estimating the impact of United States Agency for International Development child health programs

In 1995, the World Health Organization convened global partners to develop a new approach to child healthcare in developing countries, called Integrated Management of Childhood Illness (IMCI). An integrated and holistic approach to child health, IMCI aims to reduce child mortality and morbidity while promoting health and well-being of children under five years of age [18]. Shortly after development, different countries moved with varying speed to implement the recommendations of IMCI [19]. By mid-1999, 20 countries were introducing IMCI, 31 countries had introduced IMCI and were beginning to implement activities, and 12 countries had begun the expansion phase, while twenty-nine LMICs had not yet introduced IMCI [20]. In September 2000, 189 nations met at the United Nations to sign the Millennium Declaration, committing them to try to achieve the Millennium Development Goals (MDGs). MDG4 aimed to reduce child mortality by two-thirds from 1990 levels by 2015. In addition, the second half of the treatment period, from 2006 on, saw a dramatic year-over-year increase in external funding for malaria programs—including the Global Fund, World Banking, and USAID under the President’s Malaria Initiative or PMI, and other donors—in countries where it was a major cause of child mortality, primarily in Africa [21].

**Fig. 1:**
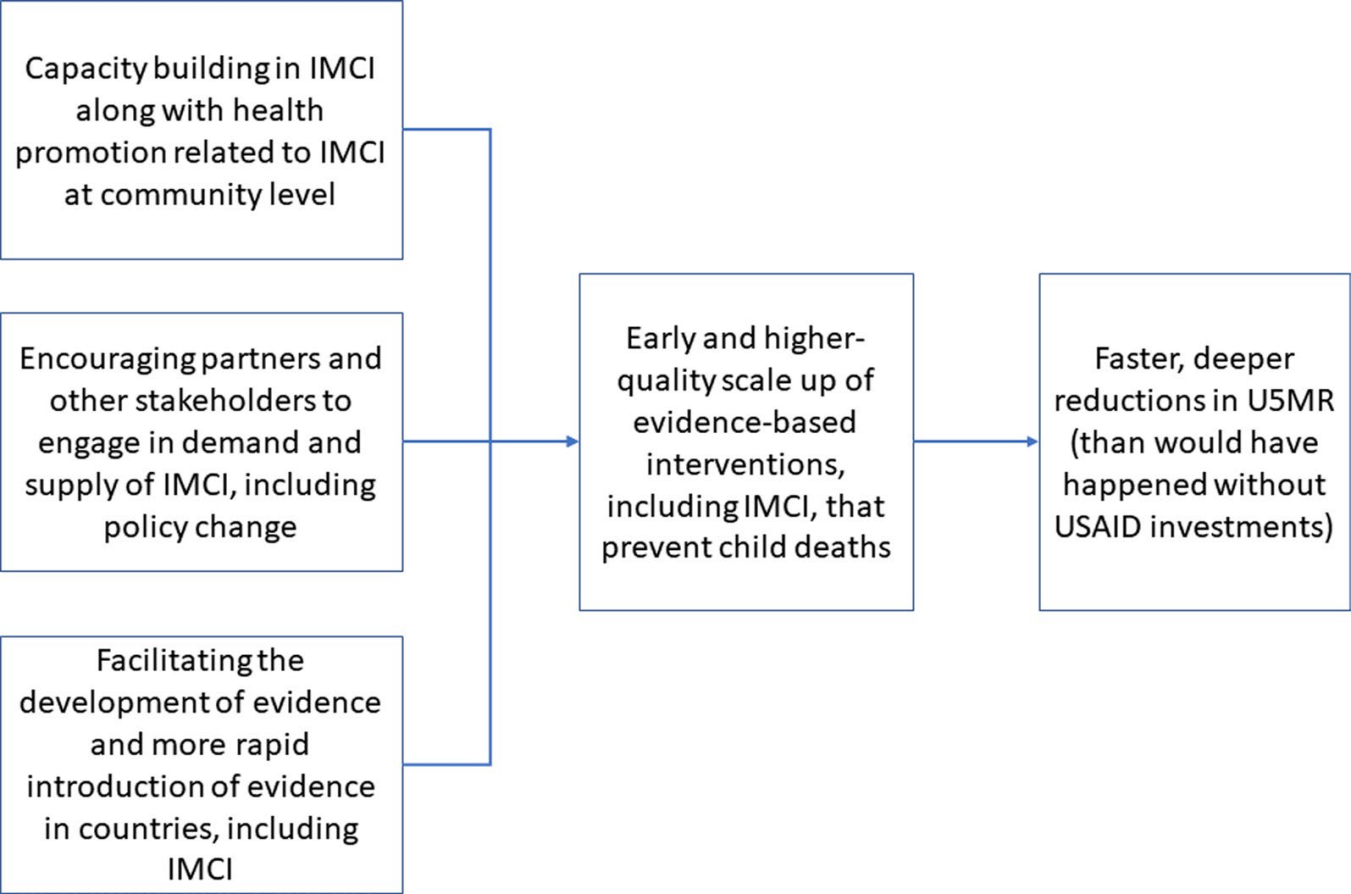
Theory of change by which child mortality reduction is faster in countries with USAID maternal and child health and malaria investments than in otherwise comparable countries

USAID was well positioned to contribute to these initiatives. To better describe here USAID’s approach to child mortality reduction, we reviewed USAID’s annual Reports to Congress on the Child Survival and Health Programs Fund from 2001–2004 as these were the early years of IMCI and the MDGs [22–25]. We reviewed the activities that USAID invested in during this period (e.g., social and behavioral change), the specific problems being addressed (e.g., treatment of diarrhea), and USAID’s approaches (e.g., quality improvement). One common attribute across USAID child health programs (including malaria-specific programs) was that activities were directed at all levels of the health system, from household level health promotion to national policy. Another common attribute was a focus on introducing and scaling up high-impact interventions for the major causes of child mortality and morbidity, including developing the evidence to support scale and quality. Most programs addressed the treatment of the sick child, including diarrhea and pneumonia, and malaria where relevant. The final common attribute was USAID support for three broad groupings of activities: developing and leveraging partnerships with the government, civil society and donors; health system strengthening; and targeting cost-effective high-impact interventions to those in most need. These common attributes across countries and other review findings are used to describe the theory of change for this analysis (Fig. 1).

We hypothesize that US investment, engagement, and partnership with countries, during the IMCI period, would accelerate reductions in child mortality in those countries, as visualized through the theory of change (Fig. 1): (1) increased capacity building and health promotion related to Child Survival (improving both supply and demand); (2) encouraging partners and other stakeholders to increase engagement with Child Survival including more rapid policy change; and, (3) facilitating development of evidence and/or more rapid introduction of evidence-based practices to reduce child mortality. These efforts would lead to earlier and more successful scale-up of Child Survival interventions, and faster under-five mortality reduction, than without these investments. More specifically, we hypothesize that under-five mortality rates (U5MR) in countries with consistent and strong USAID investment would, over the last two decades, be quantifiably lower than they would have been if USAID had not invested and engaged.

## Methods

### Synthetic control analysis method

This paper uses the synthetic control analysis (SCA) method to retrospectively quantify the impact of USAID funding on under-five mortality across several low- and middle-income countries. While other approaches also quantify impact (e.g., difference-in-difference, propensity scores), the SCA method does not require some of the critical assumptions of these other approaches [15]. The SCA method uses a nonparametric, data-driven procedure to create a control group (a synthetic control) that is similar to a treatment group in a pre-intervention period [14]. The SCA method assesses the outcomes (and predictive factors) of a group of non-treatment countries and identifies a subset of countries that are similar to the treatment countries. The SCA process assembles the counterfactual by weighting the outcomes of this subset of countries to produce a synthetic control: the outcome in the treatment countries in the absence of treatment [15]. Outcomes for the control group are then compared to the outcomes in the treatment group during the intervention period to quantify the impact of the treatment. In this paper, the units comprising the control and treatment groups are countries, the intervention is USAID investment in maternal and child health during the first 15–20 years of the IMCI initiative, and the outcome is the U5MR, as described in more detail below.

In sum, the SCA presented here provides a data-driven strategy to produce a counterfactual to enable the quantification of impact of USAID child health programs, including malaria-specific programs, under the conditions described. In this section, we describe in more detail the SCA method including assumptions, construction of the counterfactual, quantification of the impact and sensitivity analyses.

### Treatment unit

A synthetic control analysis (SCA) requires a treatment unit, a donor pool of similar units, a treatment period, an outcome variable, and a set of predictor variables to construct the synthetic control. We purposively selected countries with the highest amount of continuous USAID investments in child health during the IMCI period to make up the treatment unit. This is an example of ‘testing at the margins.” If we do not see quantifiable treatment effects in these high-investment countries, then we would not expect to see quantifiable effects in countries where USAID invested at lower levels. The authors did not expect the investment of one US dollar to have an impact, and we did not have any knowledge of a threshold beyond which investments would trigger a net positive impact; therefore, quantifying the impact in countries with relatively high levels of investment made most sense as the starting point for selecting the treatment units under this novel analysis approach for quantifying the impact of donor funding.

The selection criteria for high-investment countries that comprise the treatment unit entailed a two-step process. First, we selected countries that received continuous maternal and child health (MCH) funding during the period 1999–2016, based on the USAID’s annual Reports to Congress on the Child Survival and Health Programs Fund for 1999–2004 and later from the US Department’s Foreign Assistance Coordination and Tracking System financial reporting system from 2007–2016 (not available to the public). In total, 25 countries satisfied this condition. In the 1999–2004 period, funding of malaria programs was included in the Child Survival and Health Program funding and not available separately. From 2007–2016, MCH and malaria funds were separated and therefore, these two funds were combined to provide a consistent tracking of funds for the 1999–2016 period.

**Fig. 2:**
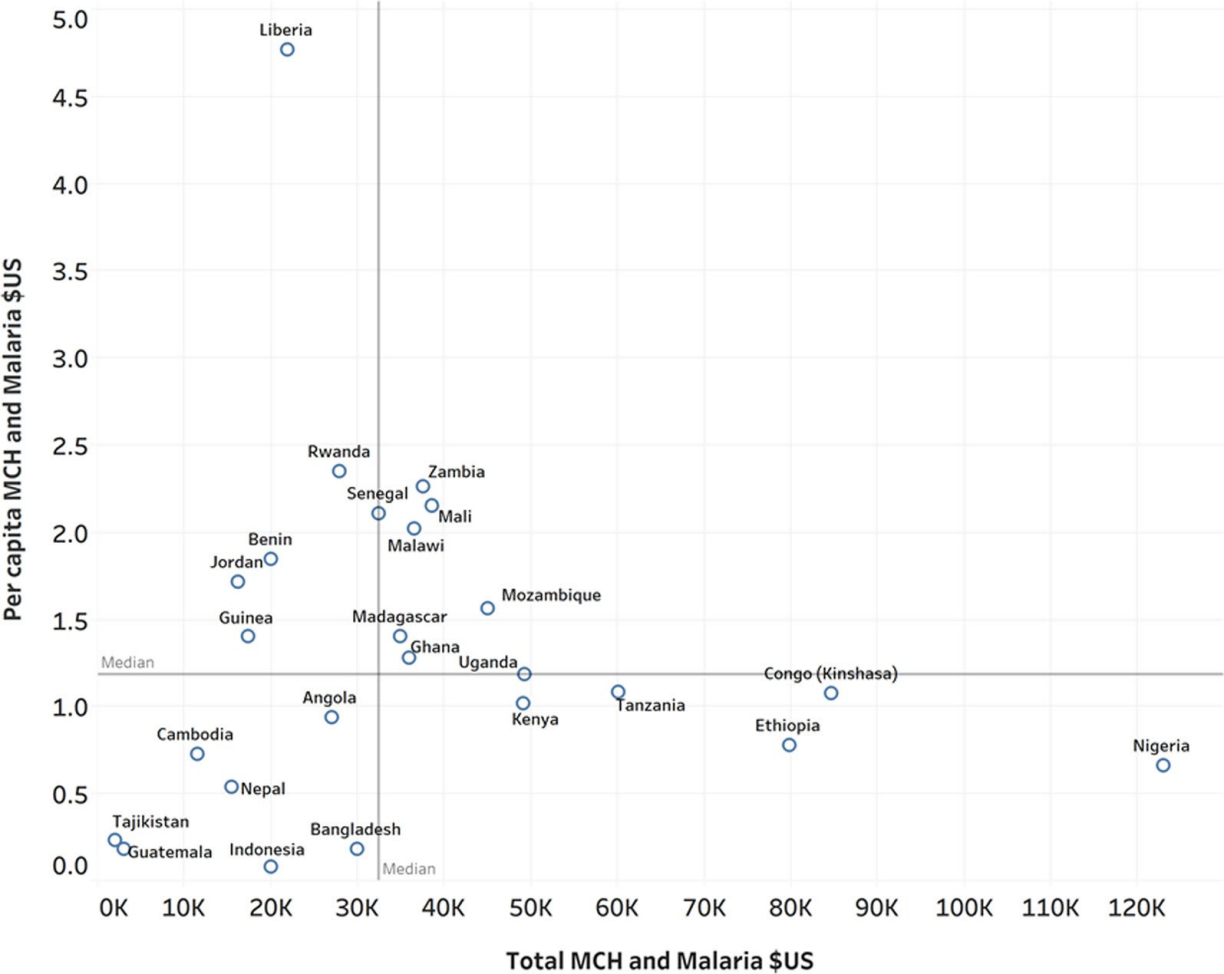
Treatment unit selection. The upper right quadrant (Quadrant 1) shows countries with above average funding in both total amount of US dollars, and per capita funding in US dollars. Funding amounts represent designated funding for maternal and child health (MCH) and for Malaria

For the second step of the process, we examined the distribution of these countries along two parameters: MCH plus malaria funding in total and per capita amounts. In Fig. 2, the axes represent the median amounts for total (*x*-axis) and per capita (*y*-axis) funding. The median annual amount received by these countries is $32.5 M and per capita amount is $1.19. From this, we identified eight high-investment countries that fall in the first quadrant of the chart, i.e., the countries that received above the median amounts on both axes: Senegal, Zambia, Mali, Malawi, Madagascar, Ghana, Mozambique, and Uganda. These eight countries make up the Treatment Unit.

Rather than use an individual country as the treatment unit, the treatment unit in this analysis was comprised of multiple treated units (the eight countries listed above), as done previously [16, 17]. We followed the alternative approach used by Lepine et al. to construct a single treated unit from the average of the outcomes and predictor variables of the eight high-investment USAID countries and then calculate the treatment effect of this single treated unit compared to the synthetic control. This was done instead of pooling the individual treatment effect of each country, since the approach we used was found by to lead to similar estimates, but with a more precise counterfactual that is less influenced by outliers [16]. The treatment unit was constructed from the eight-country yearly average of the outcome variable (U5MR) and the predictors, with the average of each variable weighted by the number of live births as estimated by the UN Population Division [26].

### Donor pool

All countries classified by the World Bank as low- or low-middle-income countries in 2016 were considered eligible to be donors. However, because SCA requires that donors not receive exposure to the treatment, countries were excluded from being in the donor pool if they received USAID financial assistance earmarked for either maternal and child health or malaria for more than half (> 9) of the sixteen years from 1999–2016. Although we originally hoped to exclude countries that received any USAID funding in the treatment period, setting this strict criterion would have eliminated almost all countries from the donor pool. Our criteria allowed 48 countries to remain in the donor pool (Additional file 1: Table S2.1a).

Another consideration with the donor pool of countries is that, as low- and lower-middle-income countries, they are likely to have received financial and technical support from other donors in the treatment period that contributed to reductions in child mortality. This leads back again to one rationale for this analysis: the need to find alternative ways to assess the impact of donor programs when there is no pure control. The potential contamination of this impact analysis from some donor pool countries that received either some USAID funds and/or other kinds of technical support for child mortality reduction is a real possibility. To account for potential differences between the donor pool and the treatment group of countries, development assistance from donors, political stability, and other factors were controlled for in this analysis (see section “Predictor variables”). Population size was also accounted for in the calculation of the outcome measure.

### Time period

We considered the treatment to have started in 1999 since many of the initial progress reports of IMCI implementation were published that year. Few, if any, countries would have started implementation sooner at any scale. Some countries in this analysis may have started IMCI later than 1999, but that is consistent with the hypothesis that USAID engagement leads to earlier and more intense implementation of new policies. The analysis scans a relatively long intervention period (1999–2016). The trends in outcome between treatment and control, during the intervention period, will reflect smaller periods of increasing and decreasing intensity in support of IMCI within the period. Choosing 1980 as the pre-intervention start year permits a long pre-intervention period (1980–1998), which helps SCA optimize its control. The year 1980 was also a good start year for the pre-intervention since data on many covariates only began to become widely available in the 1980s, due in part to the advent of the Demographic and Health Surveys. Because this analysis was initially conceptualized in June of 2017 and the dataset was compiled at that time, 2016 was chosen as the end date of the analysis.

### Dependent variable

The dependent variable is the under-five mortality rate (U5MR), since it provides an overall measure of child health and was the variable used for MDG4 [27]. The median value of U5MR is estimated annually for all countries by the UN Inter-agency Group for Mortality Estimation (UN IGME), which provides a consistent approach across countries and adjusts its estimates for shocks with significant child mortality impacts and HIV mortality, was used as the data source for the dependent variable in this analysis [27].

In 1999, the unweighted mean U5MR of the eight countries in the treatment group was 161.7, while the unweighted mean U5MR of the 48 countries in the donor pool was 80.1 [27]. This difference is expected that USAID would invest child health resources in high-mortality countries. However, the purpose of the synthetic control method is to equalize the dependent and predictor variables in the pre-intervention period (see more below).

### Predictor variables

SCA requires that the treated unit and the synthetic control be similar during the pre-intervention period when comparing across measures that may predict the outcome variable. However, child mortality reduction is multifactorial. The Success Factors Study for Women’s and Children’s Health examined over 250 indicators for data availability and potential to associate with declines in child mortality [28, 29]. It divided these many variables into 11 different policy areas (Table 1). It found that these policy areas contributed additively to child mortality reduction and that approximately half of the gains in child mortality came from improvements in coverage in the health sector (e.g., immunizations, fertility reduction), and the other half came from gains in coverage outside the health sector (water and sanitation, per capita GDP growth). Note that within these policy areas, variables were often highly correlated (e.g., between antenatal care and skilled birth attendance). For that reason, to avoid known multi-collinearity in the initial model, our initial synthetic control model included one variable from each policy area identified by the Success Factors Study, which was used as the starting point for further optimization as described below. During optimization, to avoid over-fitting the model, we did not require that the final model keep exactly one variable from each policy area, since some variables from the same policy area were only weakly correlated with each other, while others from different policy areas may have been either highly correlated to each other or of low predictive value for U5MR.

**Table 1:** Policy areas tested for synthetic cohort model

Policy area	Variables tested in synthetic cohort model
Wealth	Log GDP per capita (constant 2010 USD)
	Official development assistance per capita (USD)
Service delivery	Skilled birth attendance (%)
	Physicians per 1000 population
	Antenatal care visits (4+)
Health financing	Health expenditure per capita (2011 international $)
	Out-of-pocket health spending (% of total health spending)
Immunizations	DPT immunization (% of 12–23-month-olds)
Malaria or HIV	HIV prevalence (% 15–49 year olds)
Fertility	Total fertility rate (births per woman)
Nutrition	Stunting (% of children under 5)
Governance	Government effectiveness index
	Political stability index
	Polity score
Infrastructure	Percent of population in urban areas
	Land area
	Population density
Water, sanitation, and hygiene	Access to improved water source (%)
	Access to improved sanitation (%)
Education	Average years of female education, women aged 20–24

### Assumptions

SCA used here includes several assumptions. For accurate estimation of effects, only one unit (or one group of units) under study are treated to the intervention. The donor units cannot be exposed to the same/similar intervention, defined here as above median absolute and per capita MCH and malaria funding from USAID throughout the 2000–16 period, in addition to funding in each year of the period. Additionally, the values of the predictor variables must be comparable for both the treated and the synthetic control.

We considered the annual U5MR estimated by the IGME as the dependent variable in this paper [27]. SCA needs a form of low-rank or factor structure with additive noise, or for outcomes to follow an autoregressive process [14, 30]. The IGME estimated U5MR using the Bayesian B-splines bias-adjusted model, which uses an (hierarchical) autoregressive time series model to follow the observed changes in the data closely [31, 32]. When estimating the U5MR, the IGME data model adjusts for the errors in the observations, including the systematic biases associated with different types of data sources (viz., civil registration, sample surveys) [32]. In addition, our choices of long pre-intervention period (1980–1998) and model optimization procedure (see below) to ensure excellent pre-treatment have helped meeting the sufficient conditions for low bias in SCA [14].

### Model optimization

SCA provides an unbiased method for choosing an appropriate counterfactual for non-random treatment assignment. We iteratively added or replaced different candidate predictor variables from the model and selected the model which had the lowest root-mean-squared prediction error (RMSPE). A previous analysis that used U5MR as an outcome variable found that models with an RMSPE < 3 show a good fit between the treated unit and the synthetic control [17]. We constrained this optimization by insisting that predictors likely to confound our results (namely, polity score and total foreign aid received per capita) be included in the final model. We followed a previous SCA analysis by including three lags of the dependent variable as predictor variables [14].

### Procedure

We conducted all analyses in Stata version 14 SE using the synth command and the following code:

*synth [dependent variable] [control variables], trunit() trperiod(1999) xperiod(1980(1)1998) counit([donor countries]) nested fig allopt keep(filename.dta, replace)*

### Statistical analysis and inference

The synth procedure calculates a difference in the outcome variable between the treatment and control group in the post-intervention period, but on its own the significance of this gap is unclear. The *synth_runner* procedure in STATA permits the direct calculation of the statistical significance of the measured gap in outcomes after the intervention. *Synth_runner* performs the synthetic control procedure for the treatment unit and for each unit of the donor pool (placebos), calculating the size of the gap for each placebo each year.

Because we are testing the hypothesis that U5MR declined faster in USAID-supported countries than in similar donor countries, we use one-sided *p* values to test statistical significance. The one-sided *p* value is the number of placebos whose measured treatment gap each year was larger in the same direction as for the treatment unit, divided by the total number of placebos. Since the placebo effect may be quite large if the units were not matched well in the pre-treatment period, the measured gap for each placebo in the post-intervention period is standardized by dividing it by the pre-treatment gap size [33].

### Sensitivity analysis

We carried out several sensitivity analyses and checks to test the calculations and inferences made in the main analysis. The following analyses and checks are included in Additional file 1: Section S2:

- S2.1 Leave one out robustness check: exclusion of highest positive weight country from synthetic control
- S2.2 Analysis of Quadrant 3 countries
- S2.3 Comparing ‘nested’ and ‘non-nested’ optimization routines
- S2.4 Country-by-country analyses of Quadrant 1 countries
- S2.5 Additional uncertainty analysis using bootstrapped confidence intervals 20
- S2.6 ‘In-time’ Placebo Check
- S2.7 Pooling the treatment effects of individual treatment units
- S2.8 Comparative funding between treatment and synthetic control countries during the treatment period (1999–2016)
- S2.9 Restricting countries in the donor pool to those receiving fewer years of USAID funding
- S2.10. Repeated random assignment of eight countries in the donor pool into single control units for calculating alternative treatment effects and placebo testing
- S2.11. Results of a difference-in-difference analysis using the same donor pool and treatment units used in the main analysis

## Results

### An optimized model

**Fig. 3:**
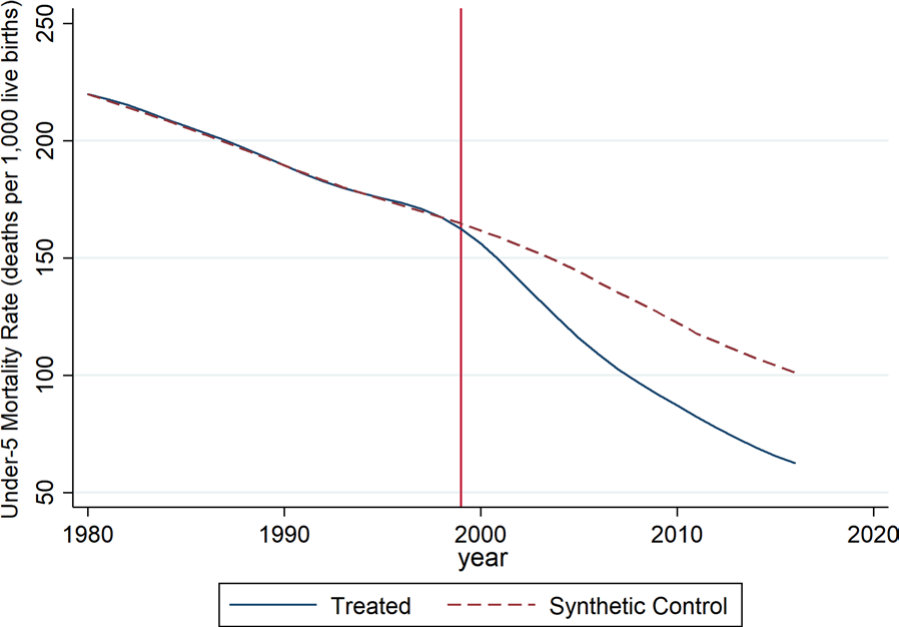
Trends in under-five mortality: Quadrant 1 (treated) versus synthetic Quadrant 1. Red vertical line reflects intervention year of 1999. Blue line represents real trend of weighted average of U5MR of countries of Quadrant 1. Red dashed line represents real trend of synthetic control U5MR

Using an iterative model evaluation process, we identified a best fit model with the lowest RMSPE of 0·5969 (Table 2) that left 10 of the original 20 predictors remaining. For example, potential predictors such as skilled birth attendance, physicians per 1000 population, and four or more antenatal care visits were removed by the model optimization to achieve the minimal difference (the RMSPE) between the treatment and control in the pre-intervention period. In this best fit model using this unbiased approach, there is close agreement between the treatment unit and the synthetic control for most predictor variables, with HIV prevalence and DPT (diphtheria, pertussis, and tetanus toxoid) vaccination coverage the main exceptions (Table 3). The synthetic control consisted of eight countries: Chad, Eritrea, Gambia, Guinea-Bissau, Mongolia, Namibia, Niger, and Swaziland/Eswatini (Table 4). This final model showed a very close agreement in U5MR between the synthetic control and treatment unit in the pre-intervention period up to 1999 (Fig. 3). Values to the left of the red vertical line reflect the pre-intervention period. The blue line shows the real U5MR trend (weighted average) of the treatment countries in Quadrant 1 of Fig. 2. Red dashed line shows the real U5MR trend of the synthetic control. There is very little difference between the blue and red lines in the pre-intervention period reflecting a very low RMSPE and a good model fit.

**Table 2:** Comparison of synthetic Quadrant 1 root-mean-squared prediction errors (RMSPE)

Synth models	RMSPE
*Outcome lags only*	
1980, 1990, 1998	2.3624
*Predictors with lags*	
TFR, stunting, HIV + 3 lags	0.9203
TFR, stunting, HIV, DPT + 3 lags	0.8477
Clean water and sanitation + 3 lags	1.0187
Logged GDP + urbanization + ODApc + 3 lags	1.7597
Polity score + 3 lags	2.0387
Logged GDP + urbanization + ODApc + polity score + 3 lags	2.2416
*Predictors without lags*	
TFR, stunting, HIV, DPT	4.5781
Clean water and sanitation	3.9344
Logged GDP + urbanization + ODApc	26.7628
*Full model*	
All 10 predictors + 3 lags	0.5969
All 10 predictors + 3 lags (without Chad)	0.6034
*Predictor year range*	
1985–1998	0.6219
1990–1998	0.6438
*Quadrant 3*	
All 10 predictors + 3 lags	0.0618
*Non-nested*	
All 10 predictors + 3 lags	1.1046

With the exception ‘Predictor year range,’ all RSMPE were generated with pre-treatment period 1980–1998

**Table 3:** Predictor means between synthetic and test case in pre-intervention period—Quadrant 1 countries

Variables*	Real	Synthetic	Mean	Std dev.
TFR	6.56	6.77	4.928765	1.681963
Stunting	49.22	48.45	35.75449	16.63775
HIV	5.10	1.32	1.552914	3.751662
DPT	47.73	30.42	58.76974	28.79853
Sanitation	17.10	17.03	45.64145	28.97808
Clean water	40.30	44.20	66.24747	22.26785
Logged GDP	6.08	6.49	7.378961	1.157757
Urbanization	23.36	23.36	41.80917	20.47601
ODA per capita	41.06	41.10	45.79208	46.53611
Polity score	− 2.90	− 2.91	− 1.25822	6.643895
Under-five mortality (1998)	167.31	167.31	103.7425	68.59552
Under-five mortality (1990)	189.46	189.43	118.0603	75.78079
Under-five mortality (1980)	219.89	219.85	147.9589	78.69472

*Ordering of these predictors is important in the nested analysis and the results can be replicated if the variables are entered in Stata syntax in the above manner

**Table 4:** Country weights in synthetic Quadrant 1

Country	Weight
Chad	0.590
Eritrea	0.031
Gambia	0.068
Mongolia	0.040
Namibia	0.076
Niger	0.077
Guinea-Bissau	0.040
Swaziland	0.078

Percent composition of synthetic control

### Quantifying the impact of USAID investments to reduce child mortality in high-investment countries

A significant divergence between the treatment unit (blue line: countries with relatively high USAID investment in child health) and the synthetic control (red line) emerges at the start of the intervention period (1999) and continues through the end of the treatment period (Fig. 3). Countries with relatively high levels of USAID investment in child health (the treatment unit) had a U5MR that was between 2 and 38 deaths per 1000 live births lower (mean = 29) than the synthetic control in the treatment period (Table 5).

**Table 5:** Post-treatment results: effects and their *p* values

Year	Estimates	Two-sided	Standardized	One-sided	Standardized
		*p* values	Two-sided *p* values	*p* values	One-sided *p* values
1999	− 2.156	0.326	0.130	0.130	0.086
2000	− 5.528	0.130	0.001	0.043	0.001
2001	− 9.991	0.065	0.001	0.001	0.001
2002	− 14.917	0.043	0.001	0.001	0.001
2003	− 19.921	0.043	0.001	0.001	0.001
2004	− 24.590	0.043	0.001	0.001	0.001
2005	− 28.302	0.065	0.001	0.001	0.001
2006	− 30.641	0.065	0.022	0.001	0.001
2007	− 32.603	0.065	0.022	0.001	0.001
2008	− 34.145	0.065	0.022	0.001	0.001
2009	− 35.084	0.065	0.022	0.001	0.001
2010	− 35.352	0.065	0.022	0.001	0.001
2011	− 35.457	0.043	0.043	0.001	0.001
2012	− 36.681	0.022	0.043	0.001	0.001
2013	− 37.445	0.022	0.043	0.001	0.001
2014	− 38.004	0.022	0.043	0.001	0.001
2015	− 38.566	0.022	0.043	0.001	0.001
2016	− 38.496	0.022	0.043	0.001	0.001

Effect size is in units of U5MR

*p* values are exact, empirical *p* values based on placebo testing. Standardization involves dividing the effect size by RMSPE

### Placebo testing and inference

**Fig. 4:**
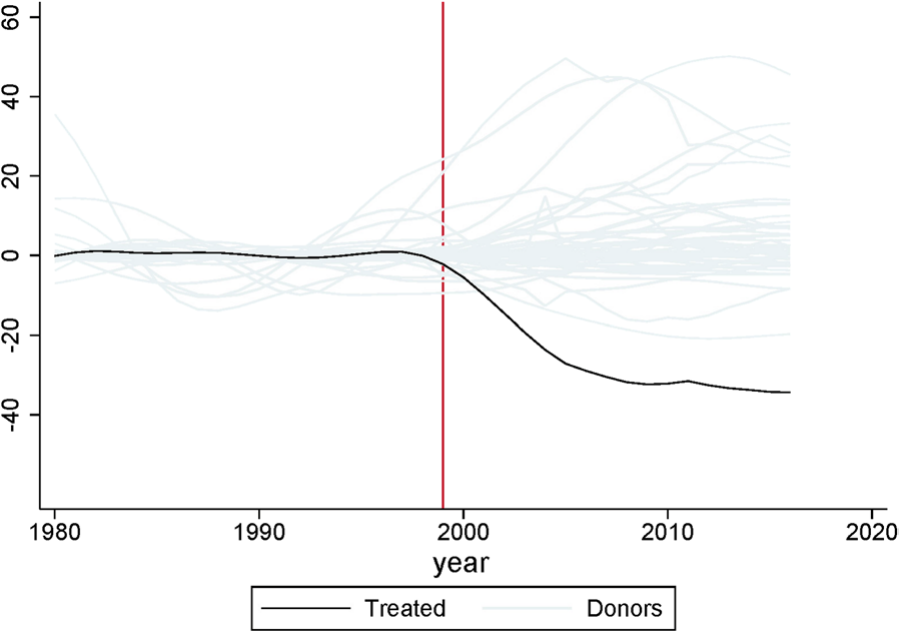
Treatment unit and 48 placebos. Red vertical line represents intervention year of 1999. All lines represent mean difference between U5MR of represented country and its synthetic control. Black line represents weighted average of Quadrant 1 countries. Gray lines represent 48 donor countries as placebos

To calculate the statistical significance of the difference between the treatment unit and the synthetic control, we check whether our estimate of the difference could be due entirely to chance using what is called a placebo test (see Abadie, et al. 2010 for more details than provided below) [34]. We check how often we would find the same or greater difference if we used each of the other countries in the donor pool at random as the treatment unit rather than the countries with high levels of USAID investment in child health that make up the treatment unit in this analysis. The placebo test provides a distribution of the U5MR differences between all the countries in the donor pool that are not part of the treatment unit and the synthetic control. If we find that the other countries in the donor pool have similar differences in U5MR compared to the synthetic control, then our inference is that there is insufficient statistical evidence that our treatment unit (countries with high levels of USAID investment in child health) has a significantly lower U5MR than the control unit. The results of the placebo testing are visualized in Fig. 4. The red vertical line represents the intervention start year of 1999. All lines represent the mean difference between U5MR of the tested country and its synthetic control. The black line represents the difference from the synthetic control and the weighted average U5MR of the original treatment unit countries (the countries in Quadrant 1 of Fig. 2 that have relatively high levels of USAID investment in child health). The gray lines represent the difference in U5MR between the 48 donor countries used as placebos and the synthetic control. The statistical significance of the placebo testing is provided in Table 5. The difference in U5MR between the synthetic control and the treatment unit (treatment unit countries with high levels of USAID investment in child health) is statistically significant in the 2000–16 treatment period (one-tailed *p* < 0.01).

### Sensitivity analyses

Additional details, tables and figures about each of these sensitivity analyses can be found in Additional file 1: Section S2.

#### S2.1 Leave one out robustness check: exclusion of highest positive weight country from synthetic control

In one sensitivity analysis, we excluded Chad from the donor pool as it had a relatively large weight among countries making up the synthetic control for a reason unclear to the authors. This was done to check whether our results would provide the same inference when a highly weighted country was removed from the donor pool. The estimated impact of high-level USAID investment in this sensitivity analysis was 19 deaths per 1000 births lower, on average, as compared to the synthetic control without Chad in the donor pool (*p* < 0.01).

#### S2.2 Analysis of Quadrant 3 countries

Another sensitivity analysis of countries with a relatively low but consistent level of USAID investments in child health (see countries in Quadrant 3 of Fig. 2) also found significant, positive impacts on under-five mortality as compared to control during the treatment period The mean difference between the treatment unit made up countries with low levels of USAID investment and the synthetic control was an under-five mortality rate that was 1.48 per 1000 live births lower. The difference was statistically significantly lower in the treatment unit for 16 of 17 years (2001–2006, *p* < 0.05; 2007–16, *p* < 0.01).

#### S2.3 Comparing ‘nested’ and ‘non-nested’ optimization routines

The SCA procedure normally returns results based on constrained quadratic optimization [15]. One option to further reduce the RMSPE is to choose nested optimization, which searches for additional combinations of control units that might reduce RMSPE. However, unpublished reports suggest that nested optimization can lead to unstable results that depend on the order variables are entered in the command line [35]. For that reason, while we aimed to select models in an unbiased way using RMSPE, we also ran a sensitivity analysis without the nested option to remove the risk of unstable results. This analysis returned similar results to the SCA model that excluded Chad (Additional file 1: Section S2.1). The average treatment effect was 17 deaths per 1000 live births lower in the treatment group than in the control with a range of 3–25 deaths per 1000, narrowing after 2010 but maintaining statistical significance throughout the treatment period (one-tailed *p* < 0.01).

#### S2.4 Country-by-country analyses of Quadrant 1 countries

We also carried out SCA for the individual countries in Quadrants 1. The results showed considerable heterogeneity, with a few showing very large reductions in U5MR compared to a synthetic control (e.g., Uganda, Zambia), many showing a small effect, and a few showing a rise in U5MR compared to a control. This variability provides support for the approach of using a more stable, single treatment unit composed of the pooled, weighted average of each country in the treatment group, to prevent the effects of outliers [34].

#### S2.5 Additional uncertainty analysis using bootstrapped confidence intervals

To examine the statistical significance of the impact of USAID investment in child health on U5MR, we estimated both nonparametric and parametric bootstrap confidence intervals for the time-varying effect estimate using an R-based application developed by Carling et al. (2016) and Li (2017) [36, 37]. The bootstrap estimation of the confidence band for the treatment unit did not cross zero in the post-treatment period, indicating that the impact of high USAID investment on U5MR was significantly different than the synthetic control in the post-treatment period (mean treatment effect = 24/1000).

#### S2.6 ‘In-time’ Placebo Check

Another check on the analysis is the ‘in-time’ placebo test that can help check on the appropriateness of the intervention period selected for the main analysis [14]. The results of the main analysis are more credible if treatment effects are observed in the main analysis treatment period but not in the ‘in-time’ placebo test period prior. We conducted an ‘in-time’ placebo by constraining the pre-treatment period to be 1980–1990, and the placebo treatment period became 1991–1998. The pre-treatment trajectory of the in-time placebo test is very close to the pre-treatment trajectory of the main analysis. However, under-five mortality trajectories of the treated unit and its synthetic control did not diverge after the placebo treatment year (i.e., 1991) as compared to the main analysis. This shows that, in contrast to main analysis SCA, the ‘in-time’ placebo has no perceivable effect. This finding lends further credibility to the selection of the treatment period in the main analysis.

#### S2.7 Pooling the treatment effects of individual treatment units

In the main analysis, the dependent and independent variables for eight countries with high USAID investment in maternal and child health are averaged (weighted by number of live births in the country) by year to create a single treated ‘average’ unit. Then the synthetic control method is used to calculate a treatment effect for the single treatment unit in comparison with a synthetic control. In this check, we do the reverse. We first apply the synthetic control method to each of the eight countries individually. We then we pool the treatment effects obtained from each treated country by calculating the mean and confidence interval of the eight estimated effects in each year [16]. We also pool 18 years of treatment effects and calculate an overall mean treatment effect with confidence interval for the entire 18-year treatment period. The treatment effects in this analysis are statistically significant and substantial (range: − 1.3 to − 22.3, mean: − 16.5) but not as large as the treatment effects in the main analysis.

#### S2.8 Comparative funding between treatment and synthetic control countries during the treatment period (1999–2016)

We checked our main analysis by observing per capita funding during the treatment period to identify whether differential funding patterns between treatment countries and synthetic control countries might explain the significant treatment effect observed in the main analysis, and as predicted by the theory of change. In this section, we explored three indicators of funding: USAID funding for maternal and child health (MCH) and malaria; (2) Net official development assistance (ODA) per capita; and (3) Total health expenditures per capita. External and/or internal non-USAID funding does not appear to explain the treatment effects of the main analysis. The pattern of USAID funding, however, is consistent with the treatment effects in the main analysis and is consistent with the theory of change. USAID MCH and Malaria funding increased in treatment countries over time relative to control countries, consistent with increasing size of the treatment effect in the main analysis, whereas the opposite was true of Net ODA or total health expenditures (see Additional file 1: Section S2.9).

#### S2.9 Restricting countries in the donor pool to those receiving fewer years of USAID funding

The donor pool in the main analysis is restricted to countries with less than 8 years of funding during the 18-year treatment period. This was done to prevent an overly small ‘*n*’ of countries leading to a potential ‘beta error’ of failing to statistically detect a true treatment effect. In this step, we check the results of carrying out the synthetic control method using more stringent restrictions: (1) countries with less than four years of USAID funding in the treatment period; and (2) countries with no USAID funding during the treatment period. In this check, we find similar treatment effects as the main analysis. In retrospect, this is not surprising as only two of the eight countries with positive weight that constitute the synthetic control received any USAID funding during the treatment period. Further, these two countries constituted only a total of 11% to the weight of the synthetic control.

#### S10. Repeated random assignment of eight countries in the donor pool into single control units for calculating alternative treatment effects and placebo testing

In the main analysis, the treatment unit is a grouped average of the eight Quadrant 1 treatment countries. The placebo test in the main analysis uses the 48 individual countries in the donor pool for placebo testing. In this sensitivity analysis, we created groups of eight countries each from those in the donor pool to act as placebos, matching the way that the eight treatment countries were grouped to create a single treatment unit in the main analysis. This was done to make the donor units used in the placebo testing more comparable to the grouped treatment unit. The 48 countries in the donor pool were randomly assigned to 48 grouped donor units of eight countries each. The treatment effects of this sensitivity analysis were consistent with the main analysis, but with a lower treatment effect size (− 19/1000 vs. − 29/1000). Standardized p-values from the grouped placebo testing were all significant (*p* < 0.001).

#### S11. Results of a difference-in-difference analysis using the same donor pool and treatment units used in the main analysis

This check was used to compare findings using a different, but commonly used analytic approach. A difference-in-difference analysis was carried out using the same eight treatment countries and the same donor pool as the main analysis using a Poisson regression model for the U5MR outcome variable. The outcome of the model supports the main analysis. Being one of the eight treatment countries equals, on average, a decrease of 2.6% in U5MR more per year in the treatment period as compared to a control country.

## Discussion

Although considered the gold standard evaluation method for drawing inferences about causality, prospective, randomized trials are often not practical for evaluation of most large-scale public health programs in low- and middle-income countries. Another program may be scaling up across a country at the same time. Or, similar programs, other than the one being evaluated, may be ongoing or completed in areas that would need to be part of a control. For these reasons, observational studies are commonly used for evaluating large-scale public health programs. To draw inferences from observational studies, however, steps must be taken to minimize potential biases. One common step to minimize bias in observational studies is the provision of a counterfactual through use of modeled controls or comparison units. This is done to simulate or model the change in the outcome variable that would have occurred in the absence of the intervention or treatment.

The synthetic control method provides a novel approach that uses a data-driven (unbiased) method to construct a counterfactual or control by creating a weighted average of the outcome variable from units in a donor pool that are most similar to the treatment unit(s). The synthetic control method then models the continuation of the trend of the pre-intervention period in both a separate treatment unit and a manufactured control unit [38]. The two trends are then compared. The difference between the synthetic control trend and the trend of the treated units is considered the treatment effect and this difference is tested for statistical significance using placebo tests or other methods. Another advantage of the synthetic control method is its ability to account for unobserved time-varying confounders that may influence the outcome variable, an advantage that other key inferential methods for analyzing observational data, such as the ‘difference-in-difference’ approach, do not have [38]. In addition, model testing procedures (placebo testing) allow the analyst to assess whether or not the control units provide an adequate counterfactual to the treatment or intervention unit(s) [15]. More traditional approaches (e.g., regression) were not considered for this analysis as these approaches do not provide sufficient methods to provide an unbiased selection of counterfactual countries, or easily allow differential weighting and contribution from the donor pool of countries.

The results of this synthetic control analysis provide a quantitative estimate of the impact on under-five mortality, on average, of USAID investments focused on child health among countries with relatively high levels of USAID investment (the treatment group) during the treatment period (2000–16): annual nominal funding of at least $32.5 M and $1.19 per capita. This period coincides with the World Health Organization’s initiative to accelerate reductions in child mortality, called the Integrated Management of Childhood Illness (IMCI) among others [39]. In the main analysis, the under-five mortality rate was more than 20 per 1000 live births lower, on average, in the treatment group as compared to the synthetic control (mean = 29, range 2–38). The difference is statistically significant (one tailed, *p* < 0.01). All 11 sensitivity analyses were supportive of the main findings, although estimates of size of the treatment effect varied. The mean treatment effect on U5MR across seven sensitivity analyses, using Quadrant 1 countries as the treatment unit, was statistically significant and ranged from − 17 to − 28 with an average of − 23 (see Additional file 1: Section S2). Most gains were achieved across these analyses in a 5-to-6-year period from the start, with some analyses leveling off and some analyses showing an increased treatment effect after that.

The inference that USAID investments have a positive impact on under-five mortality, under the conditions described above, is supported by placebo testing used to check the model used in this analysis, as well as several sensitivity analyses (see Additional file 1). For example, one sensitivity analysis (see Additional file 1: Section S2.2) with the treatment unit made up of countries with a relatively low but consistent level of USAID investments in child health also found significant, positive impacts on under-five mortality. Another sensitivity analysis (Additional file 1: Section S2.8) found that relative increases in USAID funding over time in the treatment period went along with increases in the size of the treatment effect. These findings are consistent with a dose–response effect of USAID investments in child health—that greater investment is associated with a greater treatment effect—lending additional support for a causal relationship.

There is currently insufficient information available about contextual factors and program implementation across the countries with USAID child health programs, nor comparable health indicators of coverage, impact, and cost, that would allow a better understanding the relative contribution of program interventions and context to the impact quantified in this analysis, as argued by Bryce et al. and Victora et al. [9–11]. As noted in the Introduction, this is the hope and desire for the future of program evaluation in low- and middle-income countries. The authors’ hypothesis, consistent with the high-level theory of change described above, is that a combination of the following types of USAID investments in child health contributed to the impact quantified in the analysis: (1) capacity building and health promotion around evidence-based interventions for reducing child mortality; (2) encouraging stakeholders to be engaged in scaling up and improving quality of these evidence-based interventions, including policy change; and, (3) facilitating the development of evidence for interventions that prevent child deaths, and for effective implementation of these interventions. While this analysis cannot identify the relative contribution of these approaches, we believe that substantial USAID maternal and child health and malaria investments led to countries adopting IMCI and other evidence-based MCH interventions earlier and at a higher level of intensity, scale and quality than would have happened without such investments. This assumption is supported when looking at the treatment effects by individual country that make up the treated units. The two countries with the largest difference between its trend in under-five mortality and the trend of that country’s synthetic control (Uganda, Zambia) are countries that initiated IMCI earlier and were already at the expansion phase by June 1999 [20]. (See Additional file 1: Figs. S2.4g and S2.4h). It is also important to remember that net per capita foreign aid in the pre-intervention period, among other likely confounders, was controlled for in the SCA.

## Limitations

One limitation of the SCA method is that it does not control for potential confounding variables during the treatment period. It is possible that some other factor appeared in treatment countries after 1999 besides significant USAID MCH and malaria investments and national implementation of IMCI that contributed to the observed results. Arguing against this is the strong match between the treatment unit and synthetic control in a long pre-intervention period, the long-standing USAID engagement with many of the treatment countries, and the close correlation between the introduction of IMCI and the MDGs, and the treatment initiation year. A sensitivity analysis (Additional file 1: Section S2.6) tested the timing of the treatment initiation year and did not find evidence for an earlier treatment initiation year. A related issue is that the treatment period is long (2000–16) increasing the potential for confounding (differential crises or changes in health expenditures between the treatment and the control countries). The issue of crises is discussed below. A sensitivity analysis comparing health expenditures between treatment countries and control was carried out and did not identify any advantages for the treatment countries outside of the theory of change (see Additional file 1: Section S2.8). One approach to the possibility of unmeasured, untested confounders during a long treatment period would be to give more inferential weight to the treatment effects observed in the first ‘*X*’ years of the treatment period and less weight to the later years (the right tail of the analysis) despite the statistical uncertainty intervals provided.

A substantial number of countries that received USAID funding during some years of the treatment period (but less than nine) were included in the control donor pool to maximize the size of potential controls. While excluding these countries was the preferred choice in the construction of a counterfactual, the investigators were initially concerned that excluding these countries would result in a donor pool too small to find enough countries comparable to the eight treatment countries on other measures used as predictors. To check this potential limitation in the makeup of the donor pool, a sensitivity SCA analysis (Additional file 1: Section S2.9) was performed on (a) countries in the original donor pool with less than four years of USAID funding and (b) countries in the original donor pool with no USAID funding. The results of both checks were consistent with the main analysis under both scenarios lending strength to the counterfactual used in the main analysis.

One particular SCA assumption relevant to many countries in the analysis is the absence of ‘shocks’ that might differentially affect the outcome variable—in this case child mortality rates—between the treated units and the countries making up the donor pool [38]. Such shocks might include governance crises, wars, pandemics, or natural disasters. However, the countries in quadrant 1 that make up the Treatment Unit did not experience substantial country-wide shocks in U5MR in the intervention period [27]. Countries that received considerable USAID funding and experienced considerable shocks in U5MR during the treatment period [27], and that were not included as treatment countries in the analysis (Haiti, Burma, and Indonesia), were also excluded from the donor pool because they did not get 16 continuous years of USAID funding or meet the other inclusion criteria. In addition, the source of the outcome variable, under-five mortality, is UN IGME that includes adjustments for crisis mortality in its modeled estimates [27].

The synthetic control method assumes an adequate fit between the treatment unit(s) and the synthetic control in the pre-intervention period and that the potential comparison units in the donor pool are similar to the treated unit(s) [38]. There is evidence that the pre-intervention fit in this analysis more than meets the standard (RMPSE ≤ 3) [17]. It is unlikely that the pre-intervention fit would be this good, if the treated units were not similar to the units making up the donor pool. We controlled for confounding for many cross-national differences by requiring that the synthetic control match the treatment unit on a broad range of criteria in a long pre-intervention period, including indicators of governance, health system strength, disease burden and GDP per capita. A difference in the pre-intervention period between the treatment and synthetic control groups was identified for the following predictor variables: the DPT3 immunization rate and HIV prevalence. The countries which were selected as the treatment unit, therefore, may have had stronger health systems, but also would have had a greater HIV burden putting pressure on these systems as compared to the countries that comprised the synthetic control—after unbiased selection using RMSPE minimization criteria. The possible contributions of these initial differences during the post-intervention period are unclear. The former may have helped reduce U5MR while the latter may have blunted reductions. In the end, these differences did not affect U5MR trajectory during the pre-intervention period. The higher HIV prevalence may also have attracted greater health system investment under well-financed HIV programs (e.g., the US President's Emergency Plan for AIDS Relief or PEPFAR), although PEPFAR was enacted in 2004 and the figures show the greatest year-over-year drop in U5MR occurring in the years prior to that. Nevertheless, it remains possible that some other important characteristics were not included in this analysis that may have contributed to their differential trajectories during the treatment period. However, the placebo control analysis provides some additional evidence against this.

We were also limited in this analysis by the types of available data. For example, we used the polity score as an indicator of governance environment because it was available for every country since 1980, whereas the World Bank’s governance effectiveness and political stability indices only became available in 1996. ‘Cherry-picking’ models to favor a particular outcome is a concern in SCA; we avoided this by using RMSPE as our unbiased criteria for selecting models [40].

## Conclusions

Synthetic control analysis (SCA) is a valuable addition to a range of approaches for quantifying the impact of donor programs, and for making causal inferences about the results of population level health interventions, in general, when a randomized trial is impractical. It has certain advantages over other more widely used approaches and is one of a small number of methods that may control for unmeasured time-varying confounders. Wider use of SCA, along with its dissemination and critique, will help to develop a better understanding of its strengths and limitations and optimal conditions for use [38]. The authors welcome additional attempts to replicate, publish and critique this approach.

Not unexpectedly, donor investments that complement host government child health programs appear to significantly accelerate reductions in under-five mortality when those investments are substantial in absolute amounts and per capita. A reasonable estimate of the U5MR in a country with high levels of USAID investment in maternal and child health and malaria is between 17 and 28 deaths per 1000 live births lower, after about 5 years of investment, than the same country would have experienced without this level of investment. Given the review of common USAID activities and approaches during the period of analysis, we hypothesize that a combination of the following catalytic approaches contributed to the observed impact, although the relative contributions of each cannot be assessed here: capacity building and community health promotion around evidence-based interventions; encouraging stakeholders to be engaged in scaling up and improving quality of these interventions; and facilitating intervention and implementation research in support of intervention scale and quality.

## Supplementary Information

**Additional file 1: Table S2.1a.** Donor countries. **Table S2-1b.** Quadrant 1 and Quadrant 3 countries. **Table S2-1c.** Post-treatment results: effects and their *p* values excluding Chad from donor pool. **Figs. S2.1a and S2.1b.** Treatment effect and 47 placebos (without Chad in synthetic control). **Table S2.2a.** Percent composition of Quadrant 3 synthetic control analysis. **Table S2.2b.** Post-treatment results: Quadrant 3. **Table S2.2c.** Under-five mortality predictor means in Quadrant 3. **Figs. S2.2a and S2.2b.** Synthetic control analysis and Placebo Results for Quadrant 3 countries. **Table S2.3a.** Means in pre-intervention period for predictors between synthetic control and test case. Not nested. **Table S2.3b.** Country weights in Group 1, non-nested. **Table S2.3c.** Non-nested: effects and their *p* values. **Figs. S2.3a and S2.3b.** SCA analysis for Quadrant 1 countries using non-nested option, and Placebo Results. **Fig. S2.4a.** Synthetic control analysis for Quadrant 1 country: Ghana. **Fig. S2.4b.** Synthetic control analysis for Quadrant 1 country: Madagascar. **Fig. S2.4c.** Synthetic control analysis for Quadrant 1 country: Malawi. **Fig. S2.4d.** Synthetic control analysis for Quadrant 1 country: Mali. **Fig. S2.4e.** Synthetic control analysis for Quadrant 1 country: Mozambique. **Fig. S2.4f.** Synthetic control analysis for Quadrant 1 country: Senegal. **Fig. S2.4g.** Synthetic control analysis for Quadrant 1 country: Uganda. **Fig. S2.4h.** Synthetic control analysis for Quadrant 1 country: Zambia. **Table S2.5a.** Predictor means (Web application). **Table S2.5b.** Weight of control units. **Fig. S2.5a.** Web application treatment effect by path and gap between treatment and control. **Table S2.5c.** Bootstrapped confidence intervals for the intervention effect, nonparametric and parametric models. **Fig. S2.5b.** Placebo test plot for all units (web application). **Fig. S2.5c.** Boostrap of donor pool (web application). **Fig. S2.5d.** Nonparametric estimation of treatment effect (web application). **Fig. S2.5e.** Parametric estimation of treatment effect (web application). **Table S2.5d.** Predictor means (Stata Replication). **Fig. S2.5f.** Synthetic control analysis in STATA replicating approach used in web application above. **Fig. S2.6a.** ‘In-time’ placebo test by path and gap between treatment and control groups. **Fig. S2.6b.** Nonparametric estimation of treatment effect (in-time placebo test). **Fig. S2.6c.** Parametric estimation of treatment effect (in-time placebo test). **Table S2.7.** Weighted average treatment effects in the pre-treatment (1980–1998) and treatment periods (1999–2016). **Figs. S2.8a, b and c.** Total average per capita funding and total health expenditures per capita. **Table S2.9a.** Treatment effect estimates under two scenarios: (1) donor pool limited to countries with less than four years of USAID funding in the treatment period; and (2) donor pool limited to countries with no USAID funding during the treatment period. **Fig. S2.9a.** Synthetic control analysis using a donor pool limited to countries with less than four years of USAID funding in the treatment period. **Table S2.9b.** Predictor means between synthetic and test case in pre-intervention period, Donor pool with less than four years of funding. **Fig. S2.9b.** Synthetic control analysis using a donor pool limited to countries no USAID funding in the treatment period. **Table S2.9c.** Predictor means between synthetic and test case in pre-intervention period, donor pool with no USAID funding. **Table S2.10a.** Treatment effect estimates using grouped average donor units for placebo testing. Effect size estimates are in units of U5MR. *p* values are exact, empirical *p* values based on grouped unit placebo testing. Standardization involves dividing the effect size by RMSPE. **Fig. S2.10a.** Synthetic control analysis using a donor pool of 48 groups of eight randomly selected countries. **Fig. S2.10b.** Placebo testing using a donor pool of 48 groups of eight randomly selected countries. **Table S2.11.a.** Treatment effects (incidence rate ratios) using a difference-in-difference analysis.

## Data Availability

Data and code supporting the findings can be found at the following link: https://tinyurl.com/WeissSynthControl. Additional information can be found at the following link: https://tinyurl.com/Supplementary-Materials-Synth. Additional file 1: Section S1 summarizes the utility of the synthetic control method. Additional file 1: Section S2 provides additional sensitivity analyses and additional file tables are also included.

## Abbreviations

BMGF: Bill and Melinda Gates Foundation
DAH: Development Assistance for Health
DFID: The United Kingdom's Department for International Development
DPT: Diphtheria, pertussis, and tetanus toxoid vaccine
iCCM: Integrated Community Case Management
IMCI: Integrated Management of Childhood Illnesses
LMICs: Low- and middle-income countries
MCH: Maternal and child health
MDG: Millennium Development Goals
PEPFAR: The President's Emergency Plan for AIDS Relief
RMSPE: Root-mean-squared prediction error
SCA: Synthetic control analysis
UNICEF: The United Nations Children's Fund
UN IGME: The United Nations Inter-Agency Group for Child Mortality Estimation
USAID: United States Agency for International Development
U5MR: Under-five mortality rate
WHO: The World Health Organization
