# Supplementary material for: Estimating the impact of donor programs on child mortality in low- and middle-income countries: a synthetic control analysis of child health programs funded by the United States Agency for International Development
Source: Popul Health Metr. 2022 Jan 6;20:2. doi: 10.1186/s12963-021-00278-9 (PMC8734298; doi:10.1186/s12963-021-00278-9)
Supplement: Supplementary file 1 — Additional file 1: Table S2.1a. Donor countries. Table S2-1b. Quadrant 1 and Quadrant 3 countries. Table S2-1c. Post-treatment results: effects and their p values excluding Chad from donor pool. Figs. S2.1a and S2.1b. Treatment effect and 47 placebos (without Chad in synthetic control). Table S2.2a. Percent composition of Quadrant 3 synthetic control analysis. Table S2.2b. Post-treatment results: Quadrant 3. Table S2.2c. Under-five mortality predictor means in Quadrant 3. Figs. S2.2a and S2.2b. Synthetic control analysis and Placebo Results for Quadrant 3 countries. Table S2.3a. Means in pre-intervention period for predictors between synthetic control and test case. Not nested. Table S2.3b. Country weights in Group 1, non-nested. Table S2.3c. Non-nested: effects and their p values. Figs. S2.3a and S2.3b. SCA analysis for Quadrant 1 countries using non-nested option, and Placebo Results. Fig. S2.4a. Synthetic control analysis for Quadrant 1 country: Ghana. Fig. S2.4b. Synthetic control analysis for Quadrant 1 country: Madagascar. Fig. S2.4c. Synthetic control analysis for Quadrant 1 country: Malawi. Fig. S2.4d. Synthetic control analysis for Quadrant 1 country: Mali. Fig. S2.4e. Synthetic control analysis for Quadrant 1 country: Mozambique. Fig. S2.4f. Synthetic control analysis for Quadrant 1 country: Senegal. Fig. S2.4g. Synthetic control analysis for Quadrant 1 country: Uganda. Fig. S2.4h. Synthetic control analysis for Quadrant 1 country: Zambia. Table S2.5a. Predictor means (Web application). Table S2.5b. Weight of control units. Fig. S2.5a. Web application treatment effect by path and gap between treatment and control. Table S2.5c. Bootstrapped confidence intervals for the intervention effect, nonparametric and parametric models. Fig. S2.5b. Placebo test plot for all units (web application). Fig. S2.5c. Boostrap of donor pool (web application). Fig. S2.5d. Nonparametric estimation of treatment effect (web application). Fig. S2.5e. Parametric estimation of treat [file 12963_2021_278_MOESM1_ESM.pdf]

Supplementary Material for Estimating the impact of donor programs on child mortality in low- and middle-income countries: a synthetic control analysis of child health programs funded by the United States Agency for International Development

Contents

Supplementary Section S1. Utility of the synthetic control method 2

Supplementary Section S2. Additional Sensitivity Analysis and Checks 3

S2.1 Leave one out robustness check: exclusion of highest positive weight country from synthetic control 3

S2.2 Analysis of Quadrant 3 countries 6

S2.3 Comparing ‘nested’ and ‘non-nested’ optimization routines 9

S2.4 Country-by-country analyses of Quadrant 1 countries 12

S2.5 Additional uncertainty analysis using bootstrapped confidence intervals 20

S2.6 “In-time” Placebo Check 25

S2.7 Pooling the treatment effects of individual treatment units 27

S2.8 Comparative funding between treatment and synthetic control countries during the treatment period (1999-2016) 29

S2.9 Restricting countries in the donor pool to those receiving fewer years of USAID funding 31

S2.10. Repeated random assignment of eight countries in the donor pool into single control units for calculating alternative treatment effects and placebo testing 34

S2.11. Results of a Difference-in-Difference analysis using the same donor pool and treatment units used in the main analysis 37

References 38

Supplementary Section S1. Utility of the synthetic control method

The utility of the synthetic control, as described by Abadie, Diamond, and Hainmueller[1] is the following:

- The selection of the counterfactual units is data-driven, reducing the potential for selection bias. Selection bias comes from known or unknown biases in how comparison units are selected that may systematically lead to inferences that are not true.
- The data used to identify comparison units that are a suitable match to the treatment unit(s) are transparent and the method provides a quantitative measure of the match;
- The counterfactual (the synthetic control) represents a weighted combination of non-treated/comparison units that usually provides a better match to the treated unit(s) than entire/whole single non-treated units would;
- The method provides a quantifiable measure of (a) the relative contribution of the non-treated/comparison units that are combined to create the synthetic control; and, (b) the match between the treated unit(s) and the synthetic control unit;
- These quantifiable measures allow the evaluator to judge whether or not the treated unit(s) are adequately matched with the synthetic control and therefore whether or not the synthetic control method is appropriate.
- The method goes beyond traditional difference-in-difference methods by accounting for the effects on the outcome variable by unobserved, time-varying confounders.
- A model testing method is available that produces a test statistic. The method called a “placebo test” where the synthetic control method is applied to every potential control in the donor pool. This creates a range of placebo treatment effects of placebo interventions. The exact distribution of the effects of these placebo interventions can be calculated. This allows the evaluator to compare the effect of the true intervention to the effects of the randomly generated placebo interventions and answer the following question: “How often would we obtain a treatment effect of this magnitude if we had randomly chosen the not-treated/comparison units as the treatment unit?”

Supplementary Section S2. Additional Sensitivity Analysis and Checks

S2.1 Leave one out robustness check: exclusion of highest positive weight country from synthetic control

In order to check the influence of Chad---the country with the highest positive weight among countries contributing to the synthetic control---on the main analysis, we did a sensitivity analysis without Chad in the donor pool (Supplementary Table S2.1c, Supplementary Figure S2.1a and S2.1b). This analysis compares to the main analysis in finding significant treatment effects from 2000-2016 (p. < .01). However, the treatment effects are smaller than the maximum treatment effects in the main analysis, and peak in the middle of the treatment period (instead of continuously increasing). The mean treatment effect was 19 versus 29 for the main analysis, a difference of about 10 child deaths per 1,000 live births.

Supplementary Table S2.1a: Donor Countries

Algeria	Eritrea	Namibia
Argentina	Gabon	Niger
Botswana	Gambia	Panama
Brazil	Guinea-Bissau	Paraguay
Burkina Faso	Guyana	Sierra Leone
Cameroon	Iran	South Africa
Central African Republic	Jamaica	Sri Lanka
Chad	Laos	Suriname
Colombia	Lebanon	Swaziland
Comoros	Lesotho	Thailand
Congo (Brazzaville)	Macedonia	Togo
Costa Rica	Malaysia	Tunisia
Cote d'Ivoire	Mauritania	Venezuela
Cuba	Mexico	Vietnam
Djibouti	Moldova	
Ecuador	Mongolia	
Equatorial Guinea	Morocco	

Supplementary Table S2-1b: Quadrant 1 and Quadrant 3 countries

Quadrant 1	Quadrant 3
Ghana	Angola
Madagascar	Bangladesh
Malawi	Cambodia
Mali	Guatemala
Mozambique	Indonesia
Senegal	Nepal
Uganda	Tajikistan
Zambia	

Supplementary Table S2-1c: Post-treatment results: Effects and their p-values excluding Chad from donor pool.

Year	Estimates	2-sided p-values	Standardized 2-sided p-values	1-sided p-values	Standardized 1-sided p-values
1999	-3.559	0.261	0.109	0.130	0.087
2000	-7.426	0.087	0.022	0.022	0.001
2001	-11.899	0.065	0.001	0.001	0.001
2002	-16.354	0.043	0.022	0.001	0.001
2003	-20.462	0.043	0.022	0.001	0.001
2004	-23.904	0.043	0.022	0.001	0.001
2005	-26.239	0.065	0.022	0.001	0.001
2006	-25.630	0.065	0.043	0.001	0.001
2007	-25.301	0.065	0.065	0.001	0.001
2008	-25.522	0.065	0.065	0.001	0.001
2009	-24.284	0.065	0.065	0.001	0.001
2010	-21.926	0.087	0.130	0.001	0.001
2011	-18.043	0.152	0.152	0.043	0.001
2012	-18.149	0.130	0.152	0.022	0.001
2013	-17.884	0.130	0.130	0.022	0.001
2014	-17.431	0.130	0.130	0.022	0.001
2015	-17.499	0.130	0.130	0.022	0.001
2016	-17.845	0.130	0.109	0.022	0.001

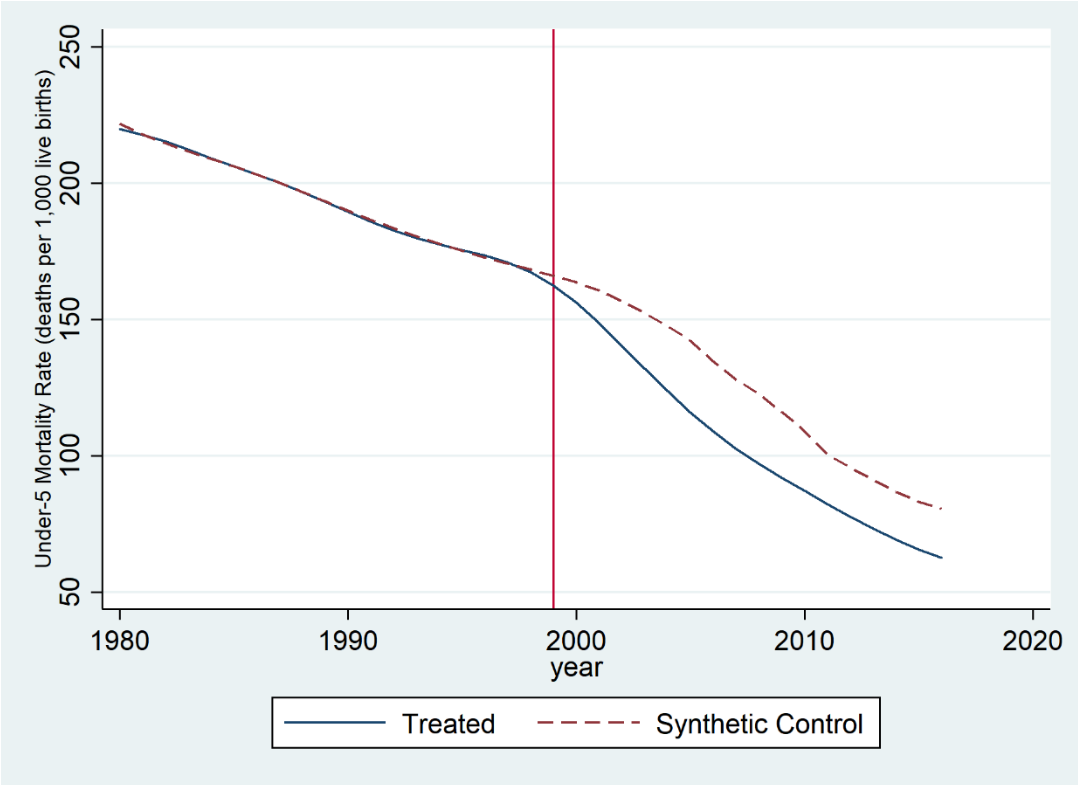

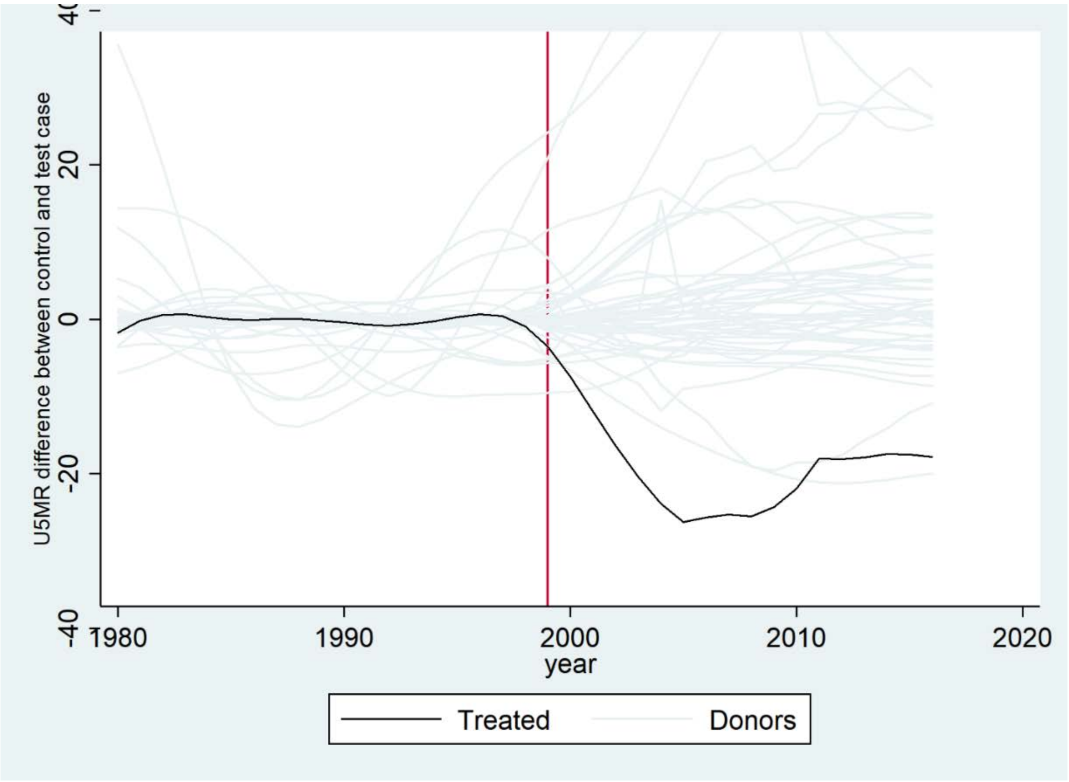

Figure S2.1a and S2.1b. Treatment effect and 47 placebos (without Chad in synthetic control)

S2.2 Analysis of Quadrant 3 countries

While we selected the treatment unit from countries in Quadrant 1 (Figure 2 of main manuscript), we also expect that the countries in Quadrant 3 should receive a positive, albeit attenuated, effect from USAID investment, since each country received some MCH or malaria funding each year of the treatment period (positive) but at below the median levels (attenuated). Therefore, we created a weighted average of the following countries: Angola, Cambodia, Nepal, Bangladesh, Indonesia, Tajikistan, Guatemala (Supplementary Table S2.1b). We considered also evaluating quadrants 2 and 4 but decided against it. Those countries with low overall total investment but high per capita investment were small countries very sensitive to destabilizing events (e.g. Liberia, Guinea, Jordan), while those countries with high total investment but low per capita investment (e.g. Nigeria, Ethiopia, Democratic Republic of Congo) are so large that USAID only operates in a limited area of those countries and there are no reliable annual data on under-five mortality in those areas. The SCA showed positive results for the treatment unit made up of Quadrant 3 countries, with statistically significant treatment effects in 16 of 18 treatment years, but the effect was attenuated compared to Quadrant 1. This finding is consistent with a dose-response relationship between intensity of USAID investment and the treatment effect (Supplementary tables S2.2a-S2.2c; Supplementary figures S2.2a and S2.2b). The treatment effect reached a maximum 3·3 deaths per 1,000 live births lower in the treatment unit by the end of the treatment period in 2016 (one-tailed p. < 0.01). In the treatment period, there were six placebos with larger reductions in U5MR than the treatment unit.

Supplementary Table S2.2a: Percent composition of Quadrant 3 synthetic control analysis.

Country	Weight
Eritrea	0.030
Comoros	0.084
Laos	0.219
Mongolia	0.395
Guinea-Bissau	0.021
Sierra Leone	0.037
Vietnam	0.207
Thailand	0.006

Supplementary Table S2.2b: Post-treatment results: Quadrant 3.

Year	Estimates	2-sided p-values	Standardized 2-sided p-values	1-sided p-values	Standardized 1-sided p-values
1999	-0.022	0.935	0.848	0.478	0.478
2000	-0.184	0.783	0.326	0.348	0.174
2001	-0.450	0.717	0.130	0.304	0.043
2002	-0.707	0.674	0.087	0.304	0.022
2003	-0.909	0.674	0.087	0.304	0.022
2004	1.628	0.652	0.043	0.370	0.043
2005	-1.244	0.696	0.109	0.283	0.022
2006	-1.365	0.739	0.109	0.304	0.022
2007	-1.466	0.696	0.065	0.261	0.001
2008	-1.569	0.696	0.109	0.239	0.001
2009	-1.698	0.717	0.087	0.239	0.001
2010	-1.877	0.696	0.087	0.217	0.001
2011	-2.106	0.696	0.087	0.196	0.001
2012	-2.425	0.630	0.065	0.152	0.001
2013	-2.751	0.609	0.065	0.152	0.001
2014	-3.042	0.587	0.065	0.130	0.001
2015	-3.197	0.565	0.065	0.130	0.001
2016	-3.260	0.587	0.065	0.152	0.001

Supplementary Table S2.2c: Under-five mortality predictor means in Quadrant 3

	**Quadrant 3**	
Variables	Real	Synthetic
TFR	4.369	4.871
Stunting	67.608	49.428
HIV	0.124	0.140
DPT	42.029	56.981
Sanitation	31.592	32.253
Clean water	65.190	53.869
Logged GDP	6.774	6.658
Urbanization	24.444	34.308
ODA per capita	11.615	33.990
Polity score	-3.604	-3.571
Under-5 mortality (1998)	87.994	87.899
Under-5 mortality (1990)	118.584	118.427
Under-5 mortality (1980)	160.039	159.777

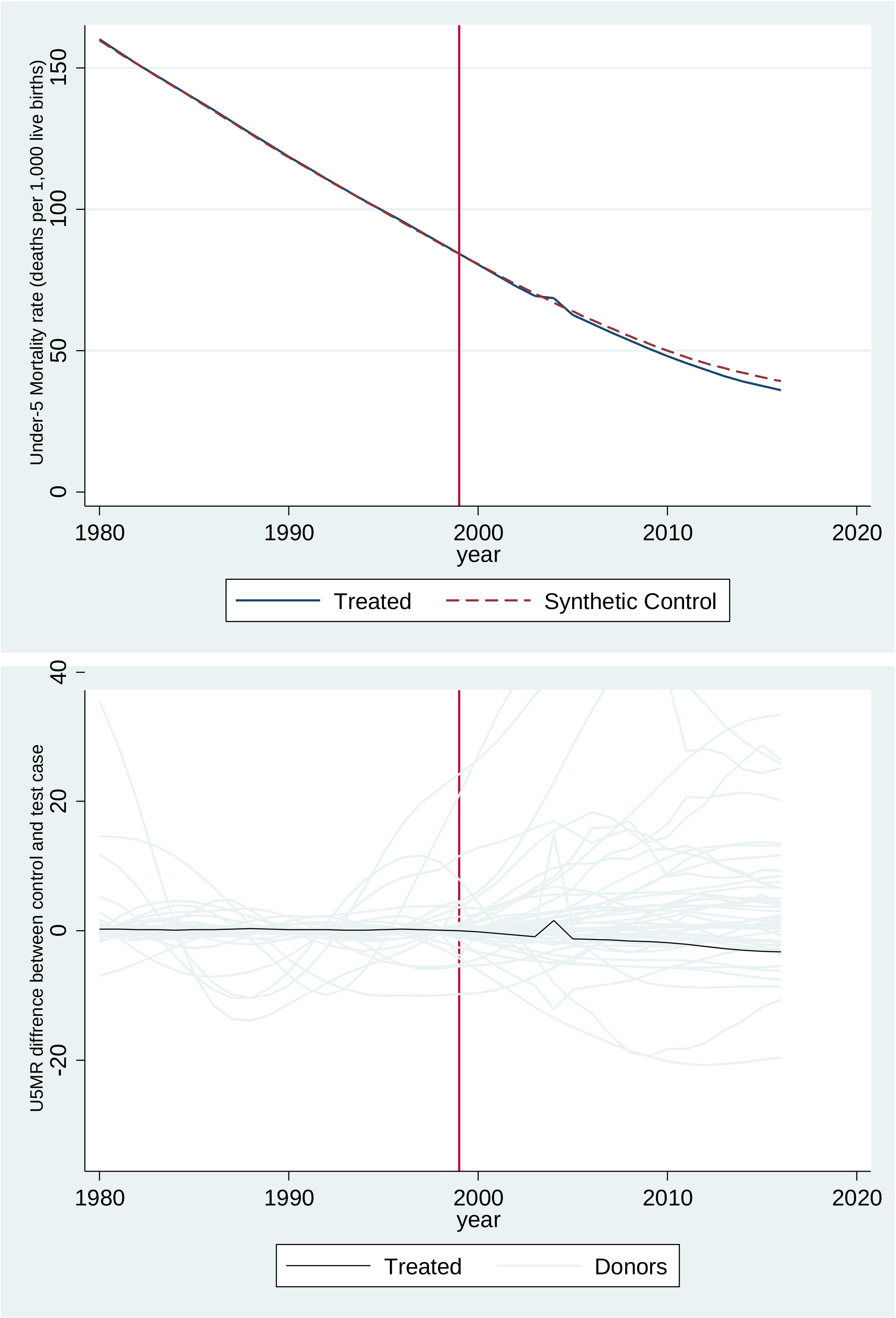

Figures S2.2a and S2.2b. Synthetic control analysis and Placebo Results for Quadrant 3 countries.

S2.3 Comparing 'nested' and 'non-nested' optimization routines

The SCA procedure normally returns results based on constrained quadratic optimization.[2] One option to further reduce the RMSPE is to choose nested optimization, which searches for additional combinations of control units that might reduce RMSPE. However, unpublished reports suggest that nested optimization can lead to unstable results that depend on the order variables are entered in the command line.[3] For that reason, while we aimed to select models in an unbiased way using RMSPE, we also ran the analysis without the nested option to remove the risk of unstable results (Supplementary tables S2.3a-S2.3c; Supplementary figures S2.3a and S2.3b). This analysis returned similar results to the SCA model that excluded Chad. The RMSPE increased to 1·10 but well below the maximum standard of 3.0. The average treatment effect was 17 deaths per 1,000 live births lower in the treatment group than in the control with a range of 3 – 25 deaths per 1,000, again narrowing after 2010 but maintaining statistical significance throughout the treatment period (one-tailed p. < .01).

Supplementary Table S2.3a: Means in pre-intervention period for predictors between synthetic control and test case. Not nested.

	**Quadrant 1**	
Variables	Real	Synthetic
TFR	6.56	6.66
Stunting	49.22	40.15
HIV	5.10	3.08
DPT	47.73	45.51
Sanitation	17.10	28.03
Clean water	40.30	42.86
Logged GDP	6.08	6.63
Urbanization	23.36	20.07
ODA per capita	41.06	46.64
Polity score	-2.90	-5.24
Under-5 mortality (1998)	167.31	167.31
Under-5 mortality (1990)	189.46	189.44
Under-5 mortality (1980)	219.89	219.81

Supplementary Table S2.3b: Country weights in Group 1, non-nested.
Percent composition of synthetic control.

Country	Weight
Eritrea	0.021
Gambia	0.121
Niger	0.345
Guinea-Bissau	0.002
Swaziland	0.372
Burkina Faso	0.139

Supplementary Table S2.3c: Non-Nested: Effects and their p-values.
Results without including nesting in the optimization procedure.

Year	Estimates	2-sided p-values	Standardized 2-sided p-values	1-sided p-values	Standardized 1-sided p-values
1999	-2.964	0.250	0.021	0.125	0.001
2000	-7.161	0.083	0.001	0.021	0.001
2001	-11.851	0.042	0.001	0.001	0.001
2002	-16.253	0.042	0.001	0.001	0.001
2003	-20.134	0.063	0.001	0.021	0.001
2004	-23.128	0.083	0.001	0.021	0.001
2005	-24.903	0.083	0.001	0.021	0.001
2006	-23.553	0.125	0.001	0.021	0.001
2007	-22.699	0.146	0.001	0.021	0.001
2008	-22.725	0.125	0.021	0.021	0.001
2009	-21.372	0.125	0.021	0.021	0.001
2010	-18.960	0.125	0.021	0.021	0.001
2011	-14.872	0.208	0.146	0.063	0.001
2012	-15.321	0.188	0.125	0.042	0.001
2013	-15.353	0.188	0.146	0.042	0.001
2014	-15.071	0.167	0.146	0.042	0.001
2015	-15.308	0.167	0.146	0.042	0.001
2016	-15.831	0.167	0.104	0.042	0.001

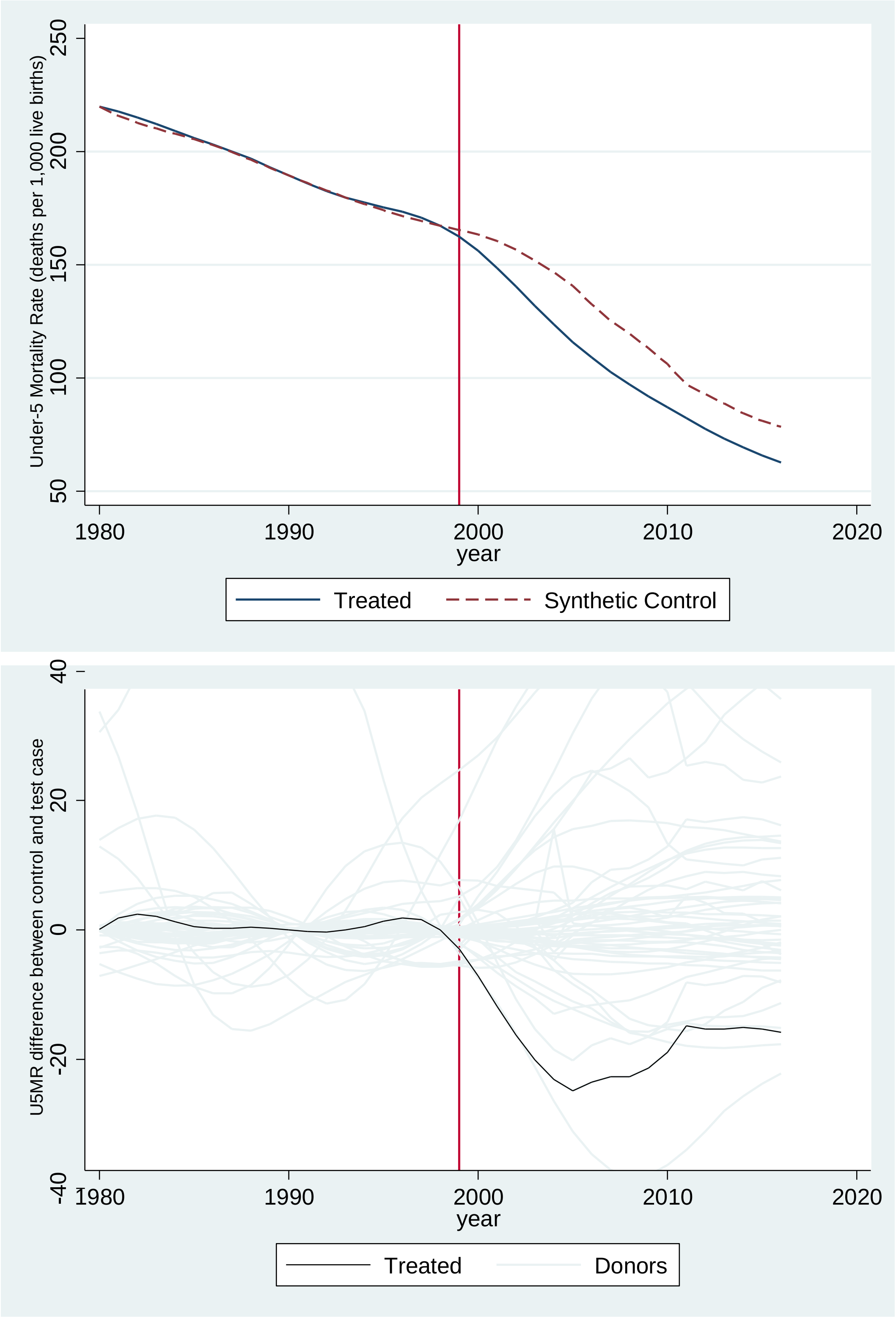

Figures S2.3a and S2.3b. SCA analysis for Quadrant 1 countries using non-nested option, and Placebo Results. This analysis was stable to order of variables, and resembles the analysis without Chad.

S2.4 Country-by-country analyses of Quadrant 1 countries

We also carried out SCA for the individual countries in Quadrants 1 (Supplementary Figures S2.4a – S2.4h). The results showed considerable heterogeneity, with a few showing very large reductions in U5MR compared to a synthetic control (e.g., Uganda, Zambia), many showing a small effect, and a few showing a rise in U5MR compared to a control. This variability provides support for the approach of using a more stable, single treatment unit composed of the pooled, weighted average of each country in the treatment group, to prevent the effects of outliers.[4]

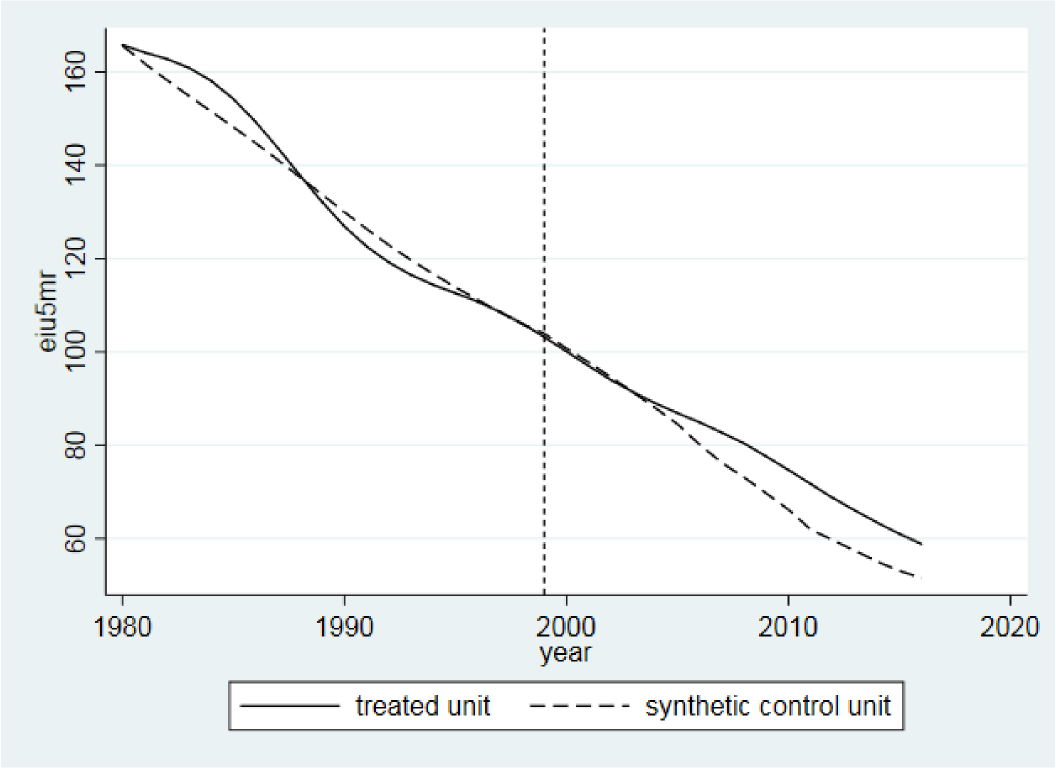

Figure S2.4a. Synthetic control analysis for Quadrant 1 country: Ghana.

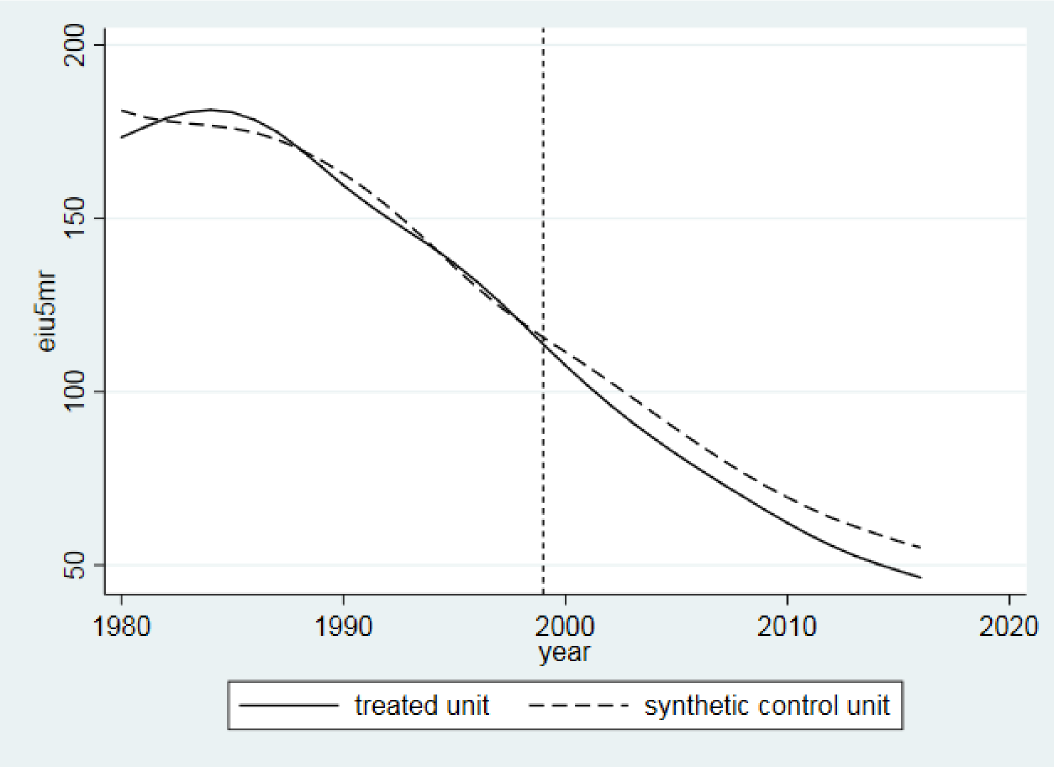

Figure S2.4b. Synthetic control analysis for Quadrant 1 country: Madagascar

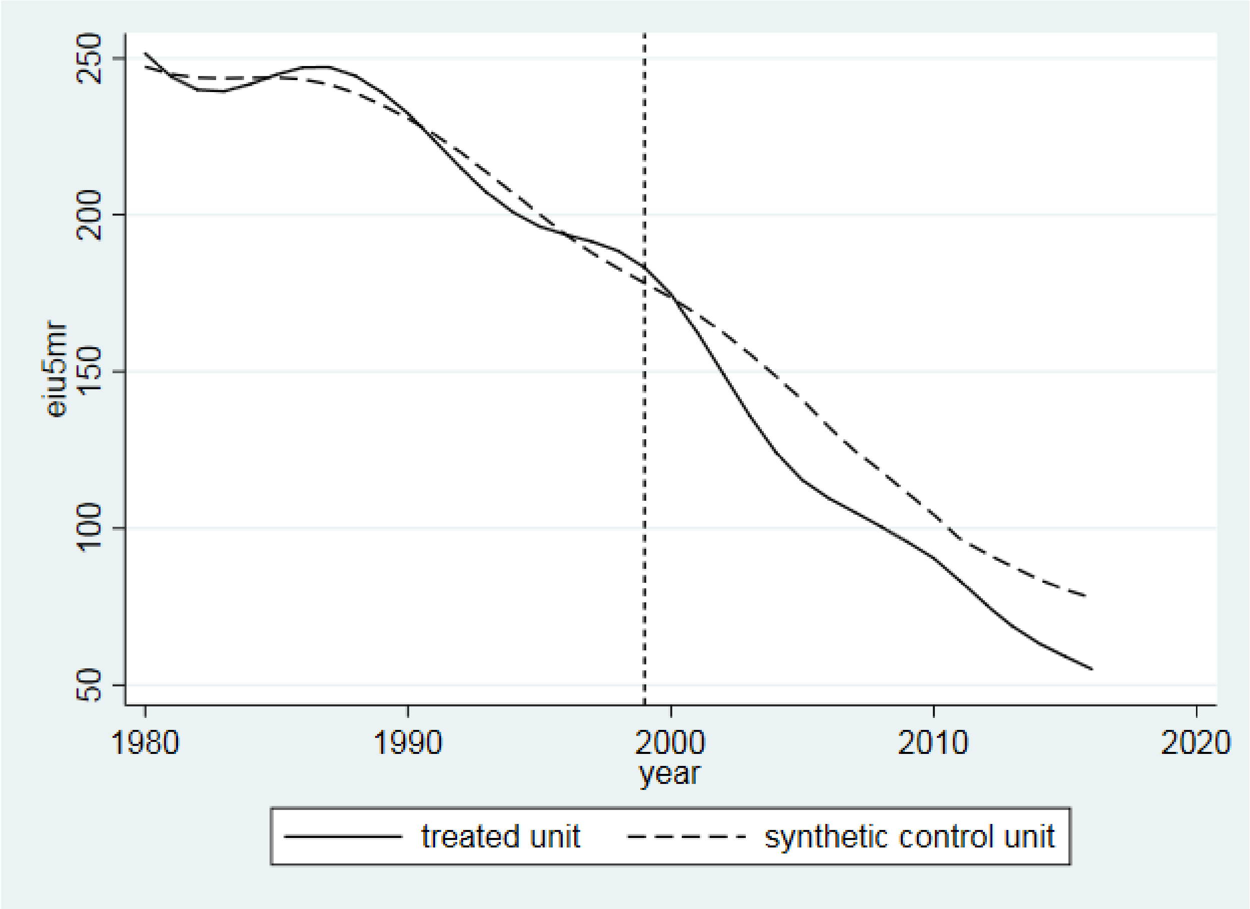

Figure S2.4c. Synthetic control analysis for Quadrant 1 country: Malawi.

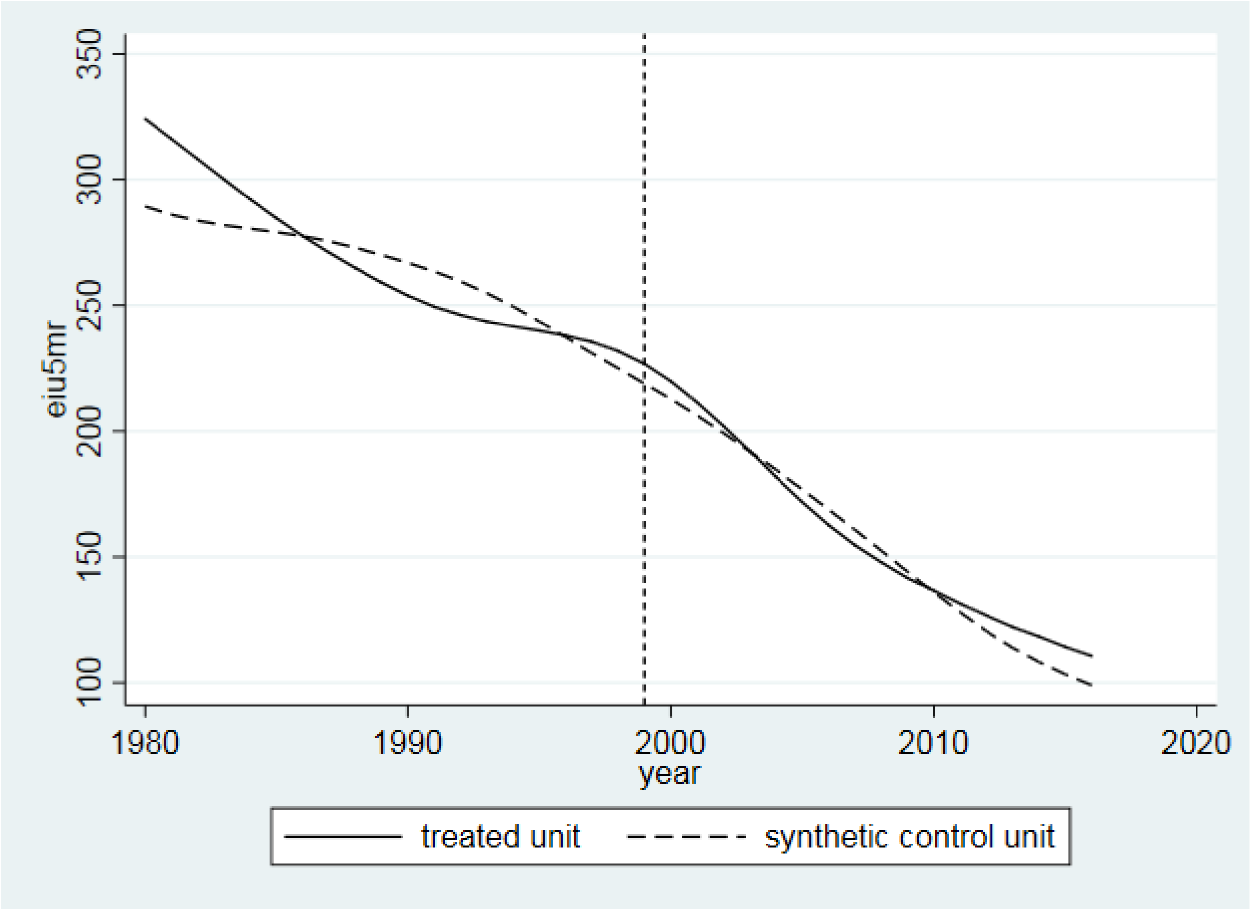

Figure S2.4d. Synthetic control analysis for Quadrant 1 country: Mali.

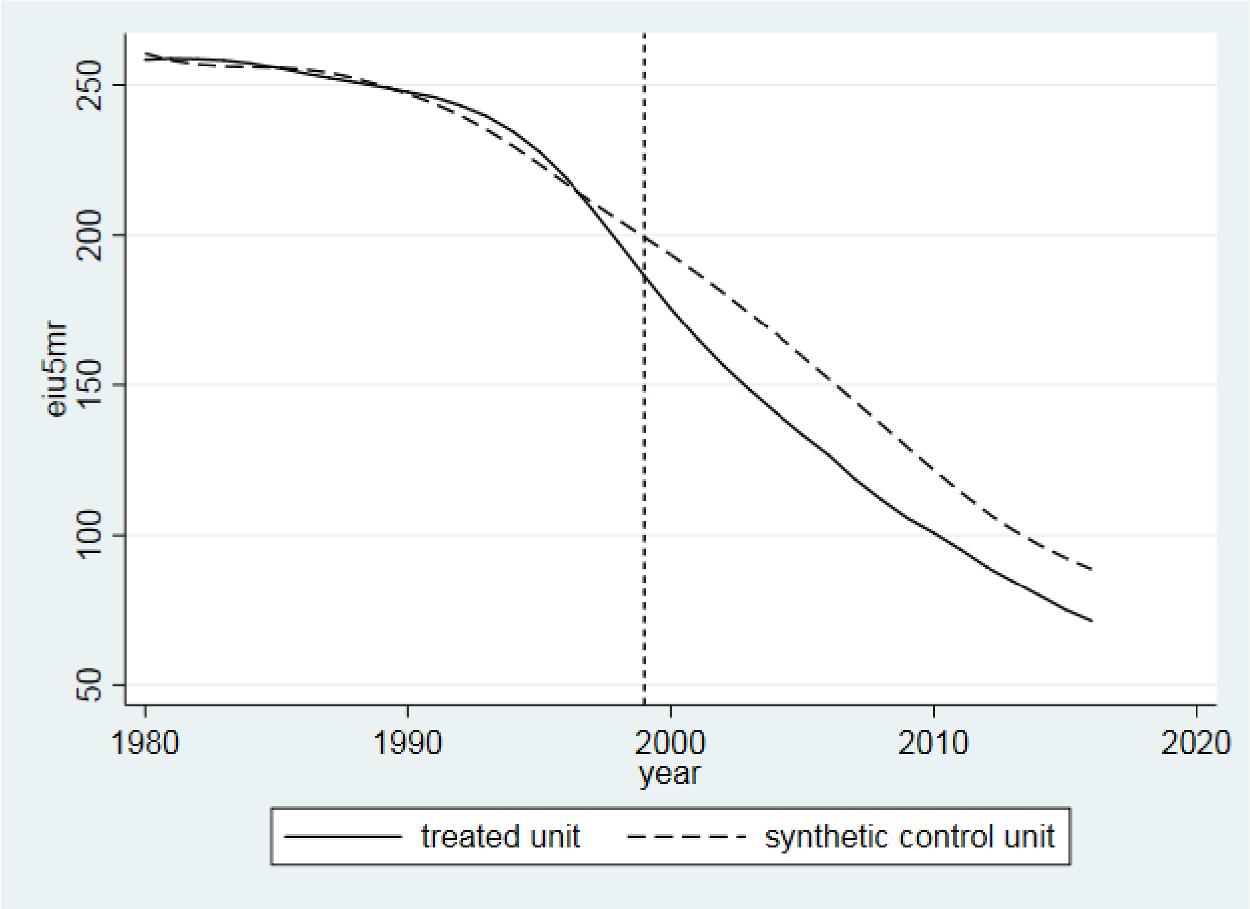

Figure S2.4e. Synthetic control analysis for Quadrant 1 country: Mozambique.

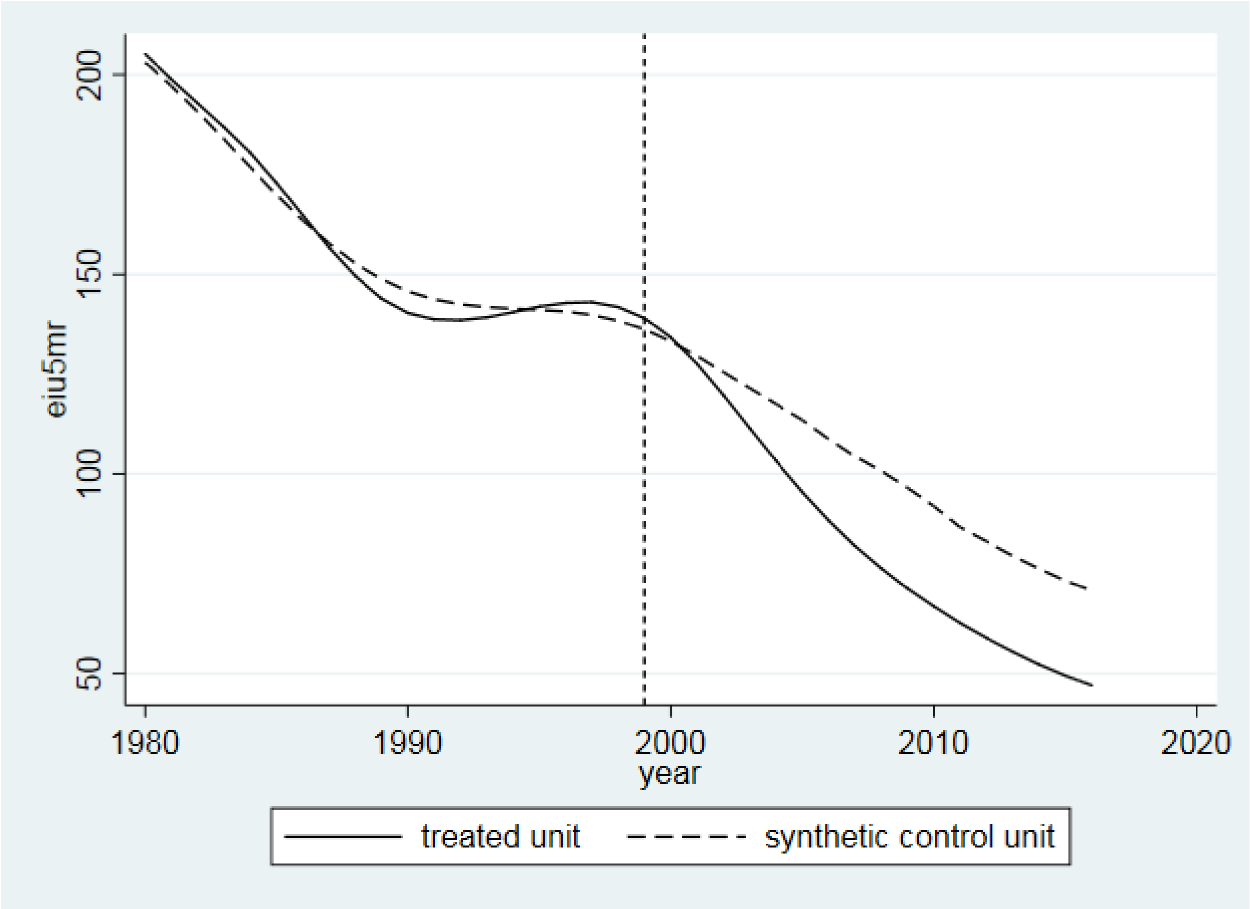

Figure S2.4f. Synthetic control analysis for Quadrant 1 country: Senegal.

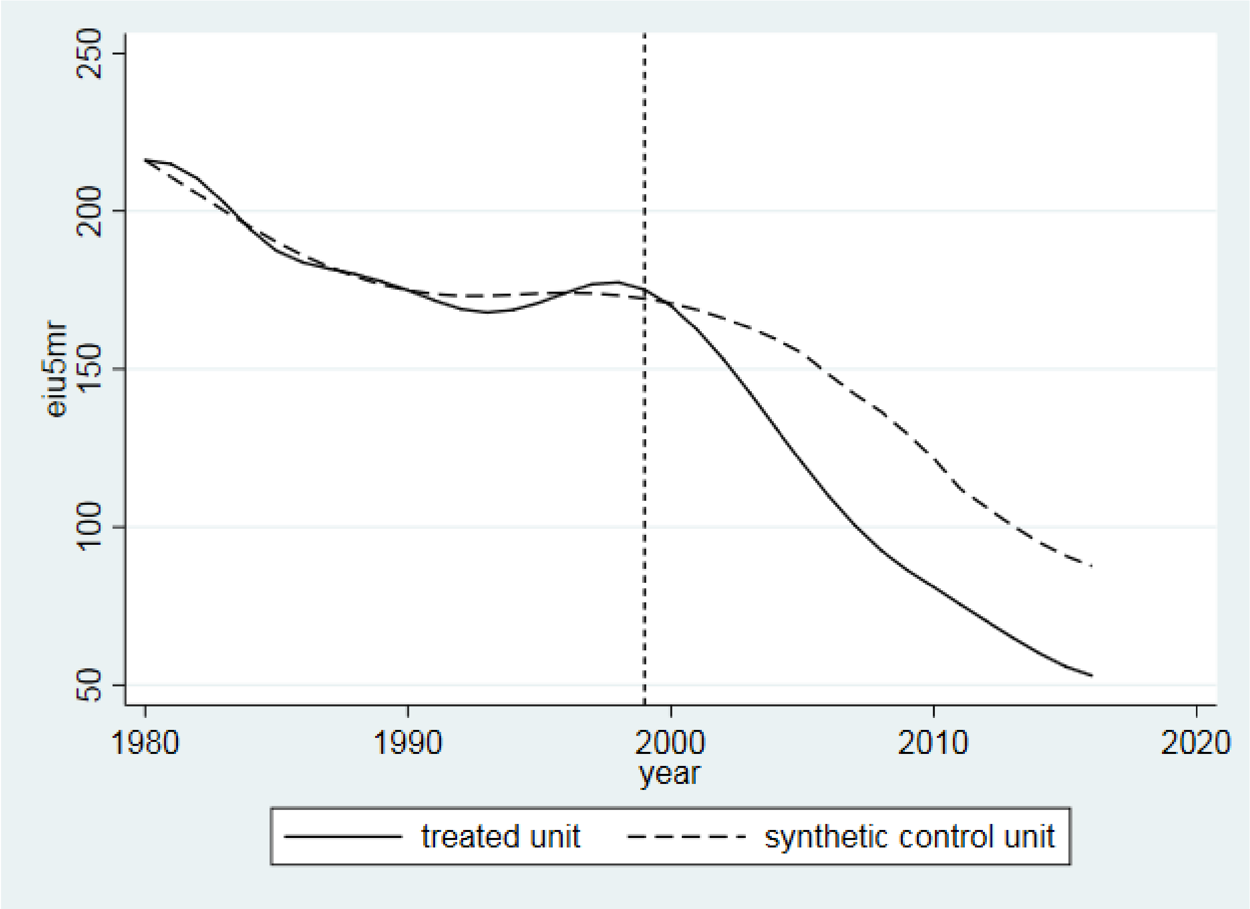

Figure S2.4g. Synthetic control analysis for Quadrant 1 country: Uganda.

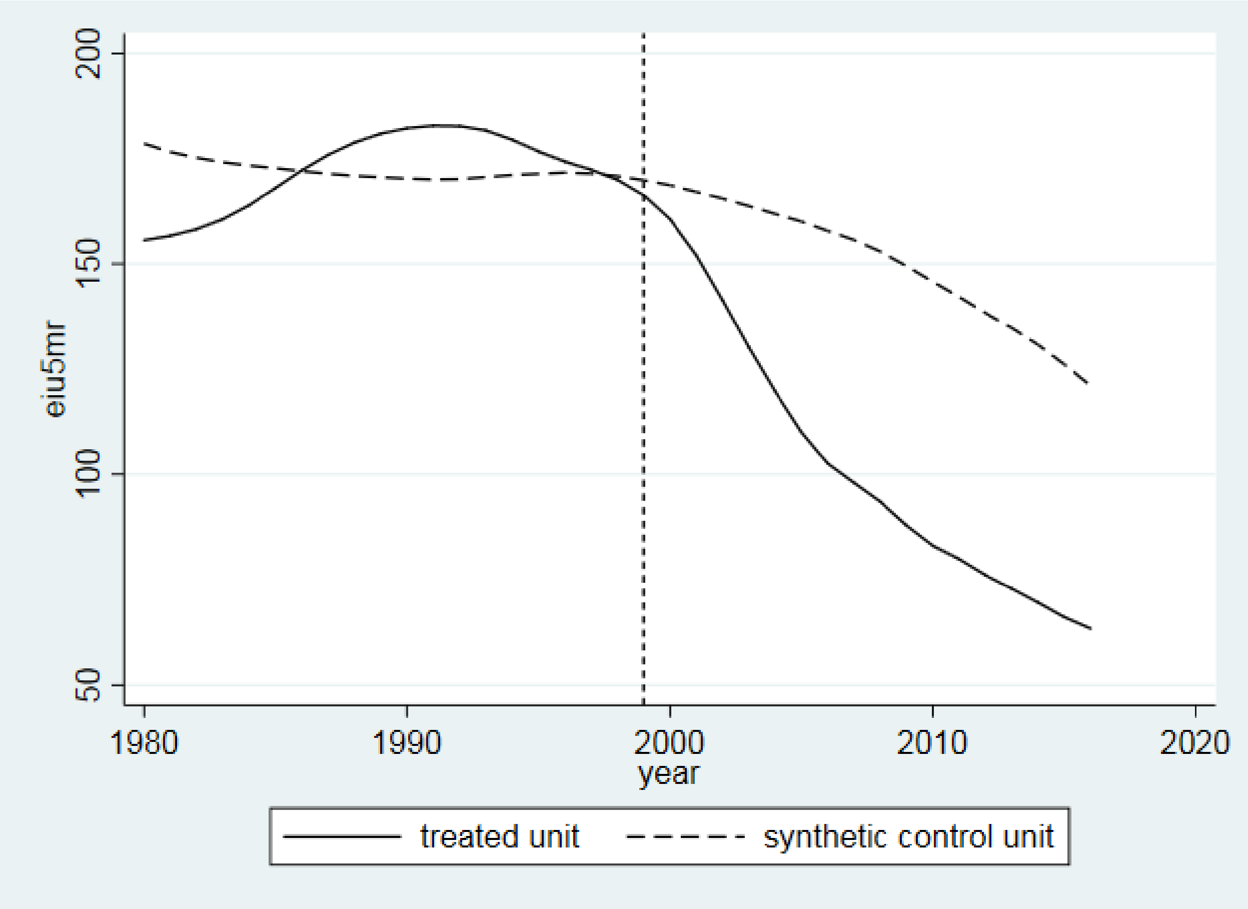

Figure S2.4h. Synthetic control analysis for Quadrant 1 country: Zambia.

S2.5 Additional uncertainty analysis using bootstrapped confidence intervals

Under this supplementary analysis, we used an R-based web-application[5] for estimating bootstrapped confidence intervals of the treatment effect. This application was created by Yujiao Li of Dalarna University, Sweden, based on the research by Carling and Li[6] and Rudholm et al. [7].

The web application uses three inference methods: first, it conducts the placebo tests suggested by Abadie, Diamond, and Hainmueller[4]; second, it estimates non-parametric bootstrap confidence intervals; and, finally, it estimates confidence intervals based on the parametric specifications of a growth model, in line with the suggestion of Carling and Li[6]. These confidence intervals are provided by the randomization of donor pool units since the unique treated unit (the average of 8 countries) is not replicable. In the non-parametric bootstrapping procedure, the application creates 200 bootstrap samples by sampling from the treated unit's donor pool units with replacement, and then applies the synthetic control method to estimate an intervention effect for each of the 200 bootstrap samples. From the resulting distribution of the intervention effect, the 0.025 and 0.975 quantiles serve as the non-parametric bootstrap confidence interval. Similarly, the parametric bootstrap confidence interval is generated by first sampling with replacement 200 times as above, and then estimating the three growth models on the new samples using the AIC to select the best-fitting model.

Following the suggestions by Abadie, Diamond, and Hainmueller[4], the web application prompted to exclude Lesotho and Niger form the donor pool based on ill-fitting placebo runs (i.e., pre-MSPE and ratio of post-/pre-MSPE, respectively). We modified the SCA accordingly and produced the following output below (see Tables S2.5a – S2.5c; Figures S2.5a – S2.5e). Both the parametric and non-parametric boostrapping methods indicate treatment effects different from zero, with an increasing treatment effect size as the intervention period progressed. This is consistent with the main analysis.

Due to inherent difference in the optimization procedure and handling of predictors (viz., special variables) between the Stata software and the web application, SCA output is consistent in direction but with smaller than the paper's main analysis. We also compared the outputs for this modified SCA in both the web application and Stata. The results from the STATA analysis---using the approach used in the web application---was similar to the findings of the web application and consistent in direction with the main analysis (see Table S2.5d and Figure S2.5f).

Web Application Results

Table S2.5a Predictor Means (Web application)

Variable	Treated	Synthetic	Sample Mean
TFR	6.56	6.57	4.58
Stunting	49.22	52	33.38
HIV	5.1	0.8	1.03
DPT	47.73	27.96	60.86
Sanitation	17.1	15.86	51.59
Clean Water	40.3	44.9	71.09
GDP (log)	6.08	6.19	7.63
Urbanization	23.36	23.24	45.86
ODA per capita	41.06	40.82	43.4
Polity Score	-2.9	-3.13	-0.74
U5MR 1980 1990 1998	192.22	192.5	94.62

Table S2.5b Weight of control units

Country	Weight
Chad	0.53
Eritrea	0.11
Guinea-Bissau	0.11
Central African Republic	0.07
Sri Lanka	0.06
Gambia	0.06

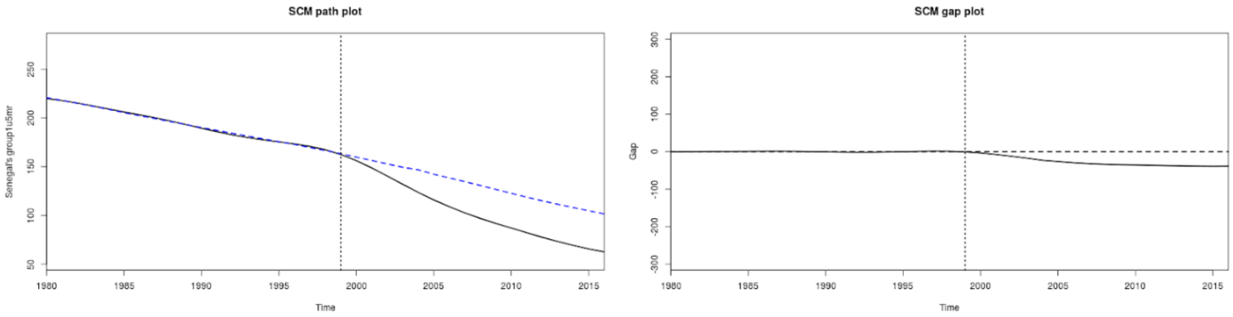

Figure S2.5a. Web application treatment effect by path and gap between treatment and control

Table S2.5c Bootstrapped confidence intervals for the intervention effect, non-parametric and parametric models

Year	Non-parametric			Parametric		
	2.50%	Mean	97.50%	2.50%	Mean	97.50%
2000	-7.1287	-3.5668	-1.0471	-14.745	-4.4229	-1.12
2001	-11.5841	-7.4318	-4.3729	-17.25	-7.9229	-3.8
2002	-16.6884	-11.9047	-8.4086	-20.4	-12.3881	-7.965
2003	-22.0137	-16.4395	-12.5571	-24.045	-16.951	-13.0925
2004	-27.7107	-20.8771	-15.6985	-27.87	-20.8026	-16.1175
2005	-31.4643	-24.0239	-18.3107	-31.4775	-23.7245	-17.5325
2006	-34.8826	-26.3702	-19.7783	-34.575	-25.8403	-17.765
2007	-37.6301	-28.1281	-19.9272	-36.675	-27.3468	-17.845
2008	-39.5279	-29.1205	-19.397	-37.64	-28.4171	-17.8725
2009	-40.9342	-29.6227	-18.6537	-38.425	-29.1771	-17.8775
2010	-41.7528	-29.5541	-17.5523	-39.5325	-29.7187	-17.88
2011	-42.7572	-29.7916	-17.0391	-40.505	-30.1058	-17.88
2012	-43.8314	-30.319	-16.7881	-41.21	-30.3848	-17.88
2013	-44.6587	-30.6575	-16.5901	-41.73	-30.5858	-17.88
2014	-45.2733	-30.9457	-16.5422	-42.1025	-30.7326	-17.88
2015	-45.5307	-31.1817	-16.618	-42.375	-30.839	-17.88
2016	-45.1508	-30.8711	-16.4941	-42.575	-30.9171	-17.88

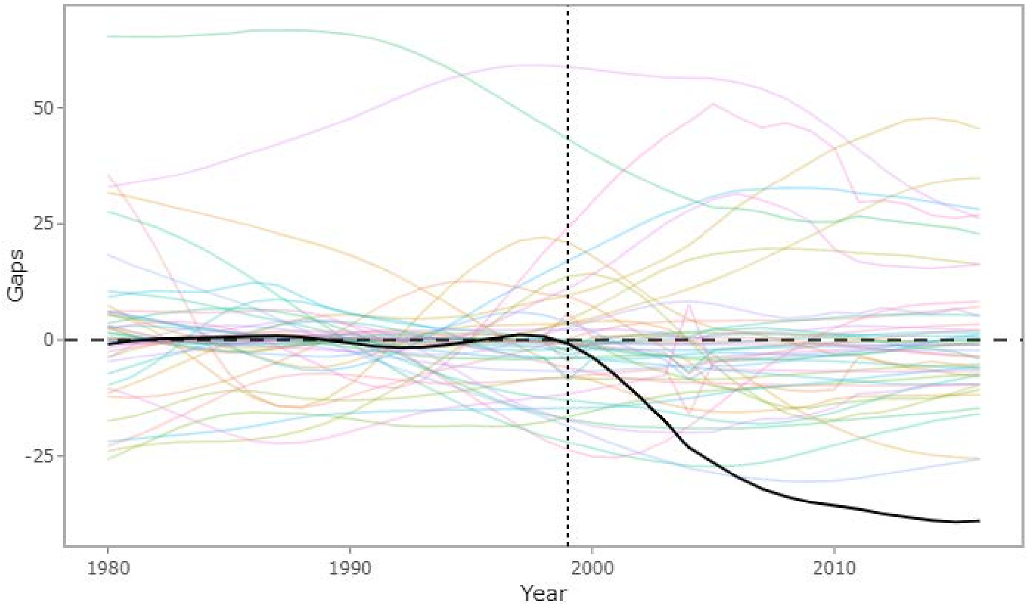

Figure S2.5b Placebo test plot for all units (web application)

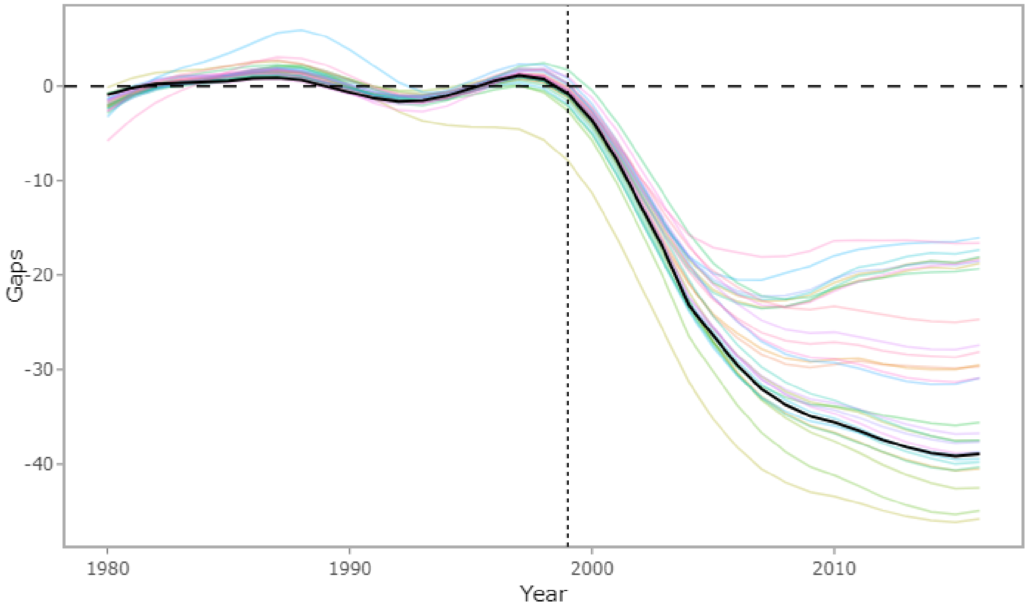

Figure S2.5c Boostrap of donor pool (web application)

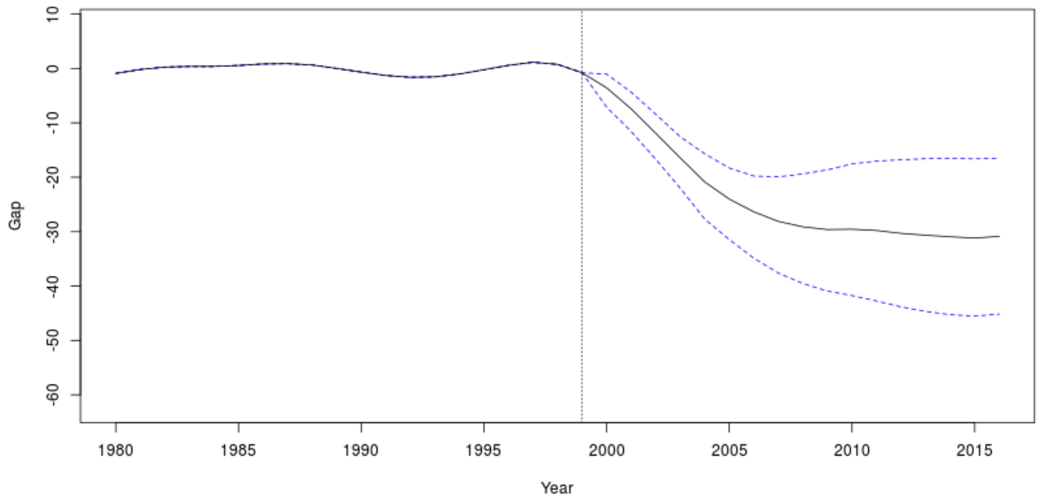

Figure S2.5d Non-parametric estimation of treatment effect (web application)

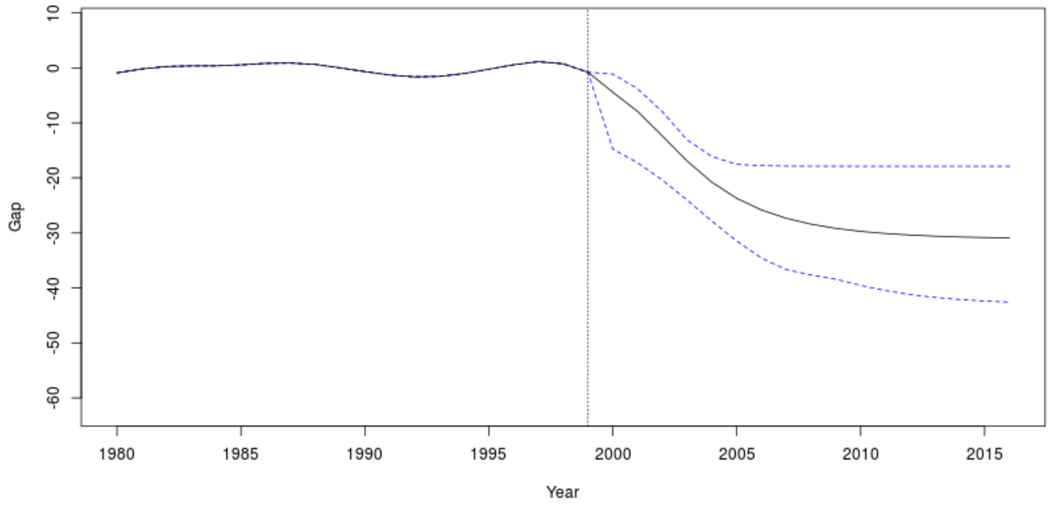

Figure S2.5e Parametric estimation of treatment effect (web application)

Replication of web application approach using STATA (desktop)

Table S2.5d Predictor Means (Stata Replication)

Variable	Treated	Synthetic
TFR	6.56	6.63
Stunting	49.2	50.64
HIV	5.1	0.96
DPT	47.73	28.74
Sanitation	17.1	17.08
Clean Water	40.3	46.94
GDP (log)	6.08	6.16
Urbanization	23.36	23.34
ODA per capita	41.06	41.00
Polity Score	-2.9	-2.9
U5MR 1998	167.3	166.7
U5MR 1990	189.5	180.3
U5MR 1980	219.9	221.2

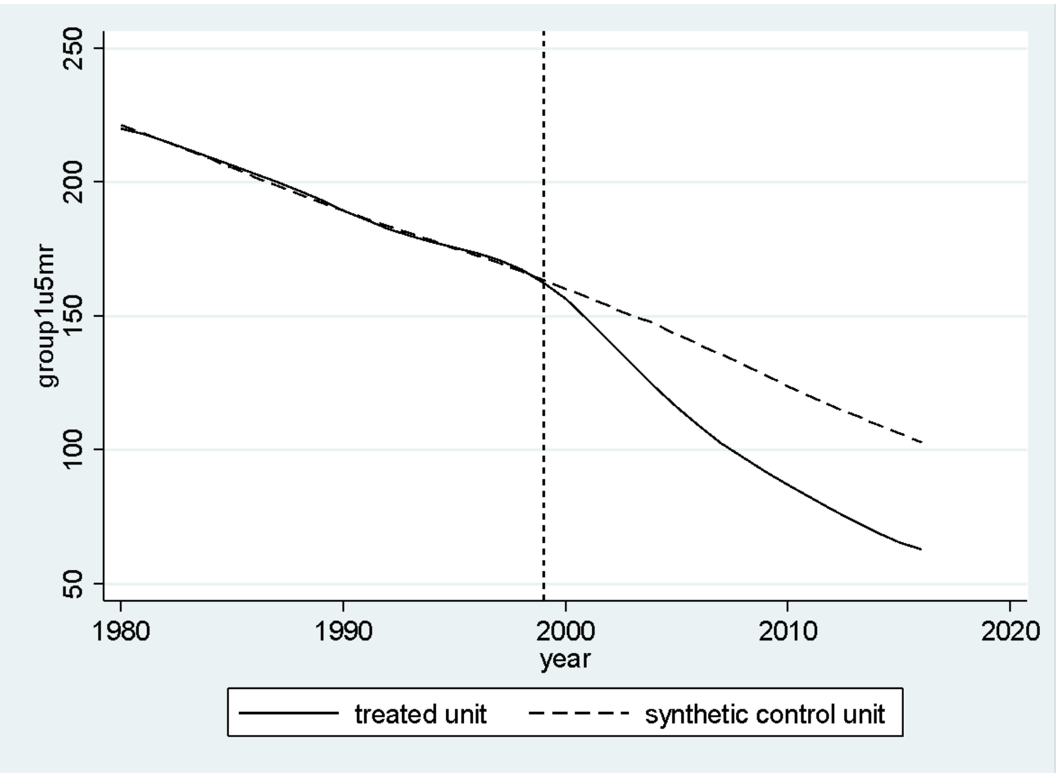

Figure S2.5f. Synthetic control analysis in STATA replicating approach used in web application above.

S2.6 “In-time” Placebo Check

Another check on the analysis is the "in-time" placebo test that can help check on the appropriateness of the intervention period selected for the main analysis. In this test, a new treatment period is designated that is before the treatment time used in the main analysis. As compared to other placebo tests, here the treatment is reassigned not across units (i.e., countries) but in time. The test checks whether any treatment effects are seen prior to the treatment period of the main analysis and evaluates how likely it is to obtain results of the magnitude that we obtain in our main analysis when we apply the synthetic control analysis in a sample period before the actual treatment year[1].The results of the main analysis are more credible if treatment effects are observed in the main analysis treatment period but not in the ‘in-time’ placebo test period prior.

We conducted an ‘in-time’ placebo by reassigning the treatment year the year 1991, eight years earlier than in the main analysis. Thus, the pre-treatment period was constrained to be 1980-1990. We also lagged our predictors variables accordingly to an earlier point in time. The figures below (S2.6. – S2.6c) display the results of our in-time placebo study. The pre-treatment trajectory of the in-time placebo test is very close to the pre-treatment trajectory of the main analysis. However, under-5 mortality trajectories of the treated unit and its synthetic control did not diverge after the placebo treatment year (i.e., 1991) as compared to the main analysis: the 95% confidence intervals (both parametric and non-parametric) overlap zero. This shows that, in contrast to main analysis SCA, the ‘in-time’ placebo has no perceivable effect. This finding lends further credibility to treatment period of the main analysis and the results.

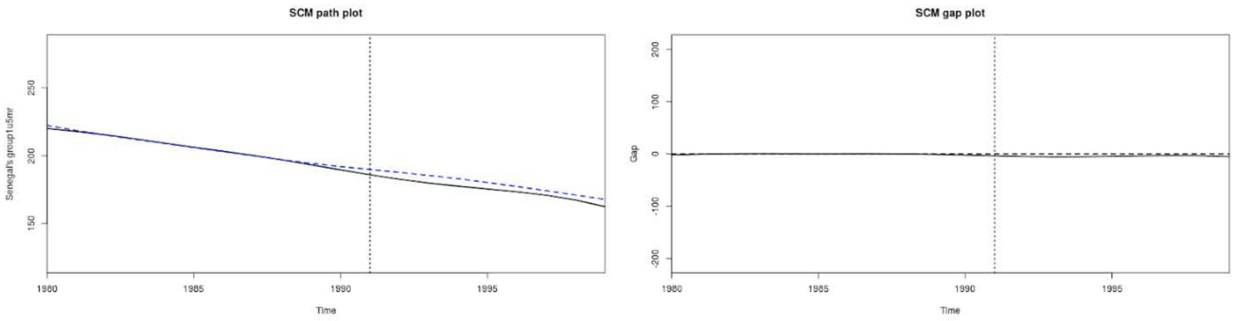

Figure S2.6a. ‘In-time’ placebo test by path and gap between treatment and control groups

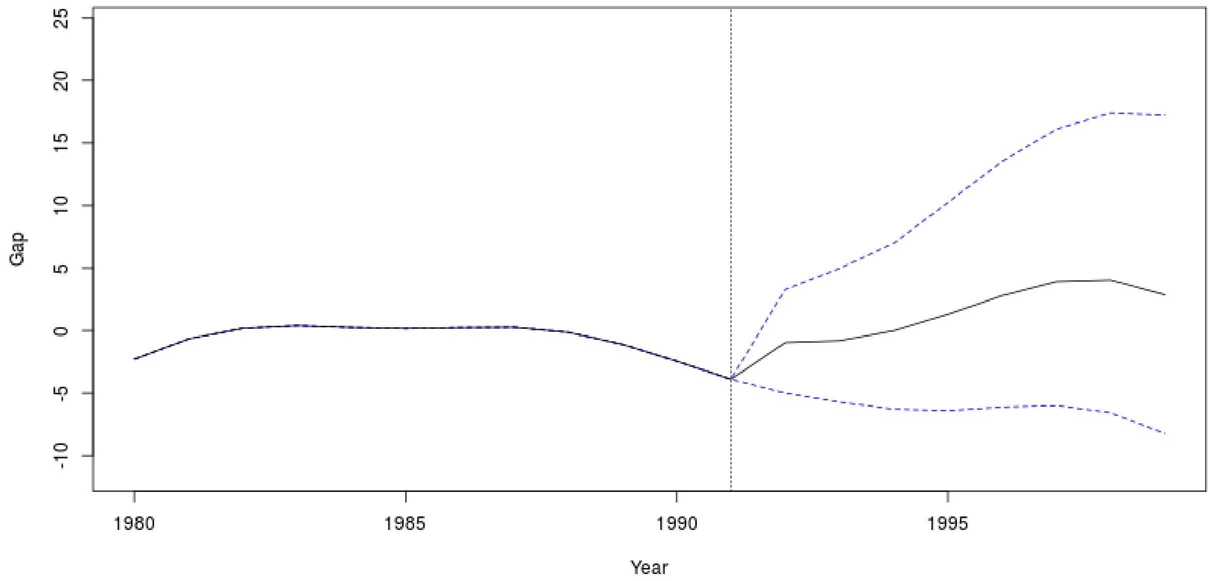

Figure S2.6b Non-parametric estimation of treatment effect (in-time placebo test)

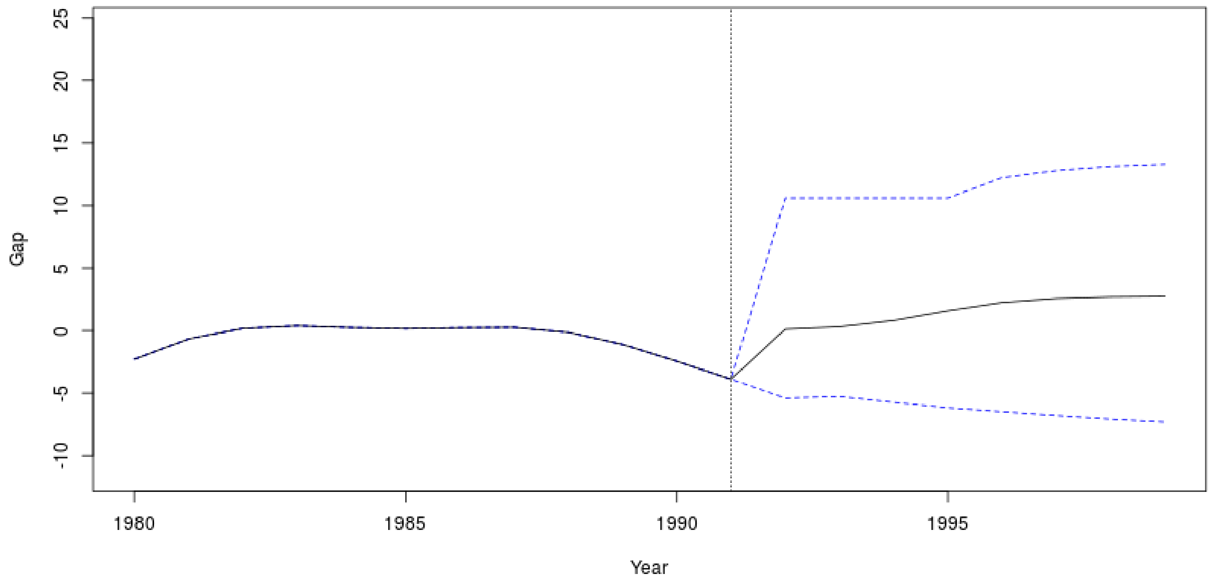

Figure S2.6c Parametric estimation of treatment effect (in-time placebo test)

S2.7 Pooling the treatment effects of individual treatment units

In the main analysis, the dependent and independent variables for eight countries with high USAID investment in maternal and child health are averaged (weighted by number of live births in the country) by year to create a single treated 'average' unit. Then the synthetic control method is used to calculate a treatment effect for the single treatment unit in comparison to a synthetic control. In this check, we do the reverse. We first apply the synthetic control method to each of the eight countries individually. We then we pool the treatment effects obtained from each treated country by calculating the mean and confidence interval of the eight estimated effects in each year weighted by the number of live births in each country by year using the STATA '*ci*' command.[8] We then pool 18 years of treatment effects and calculate an overall mean treatment effect with confidence interval for the entire 18-year treatment period.

In comparison to the main analysis, the treatment effects are also statistically significant ($p < .05$, 1-sided) in the years 2000 to 2016. The treatment effects in this analysis are substantial (range: -1.3 to -22.3, mean: -16.5) but not as large as the treatment effects in the main analysis (range: -2.2 to -38.6, mean: -29). The treatment effects in this analysis are closer to the treatment effects in "leave one out" sensitivity analysis that excluded Chad with the highest positive weight from the donor pool (range: -3.6 to -26.2, mean: -18.9) where treatment effects increased year over year until the middle of the treatment period when the effects began to get smaller, stabilizing around -17. This closer match to the 'leave one out' analysis is not surprising as the donor countries with positive weight would vary in each of the eight individual SCA analyses in this test and not necessarily include Chad.

Table S2.7 Weighted Average Treatment Effects in the Pre-Treatment (1980-1998) and Treatment Periods (1999-2016)

Period	Year	Obs	Mean	Std.	95% conf. interval	
Pre-treatment	1980	8	2.38	5.37	-10.31	15.07
Pre-treatment	1981	8	3.91	4.79	-7.43	15.25
Pre-treatment	1982	8	4.57	4.33	-5.67	14.82
Pre-treatment	1983	8	4.45	3.87	-4.69	13.59
Pre-treatment	1984	8	3.91	3.44	-4.22	12.03
Pre-treatment	1985	8	3.32	2.98	-3.73	10.37
Pre-treatment	1986	8	2.92	2.33	-2.59	8.43
Pre-treatment	1987	8	2.62	1.61	-1.18	6.41
Pre-treatment	1988	8	2.04	1.00	-0.32	4.41
Pre-treatment	1989	8	1.06	0.81	-0.85	2.98
Pre-treatment	1990	8	-0.13	1.11	-2.74	2.49
Pre-treatment	1991	8	-1.27	1.62	-5.11	2.56
Pre-treatment	1992	8	-2.08	2.15	-7.16	3.00
Pre-treatment	1993	8	-2.27	2.54	-8.28	3.73
Pre-treatment	1994	8	-1.78	2.61	-7.95	4.39
Pre-treatment	1995	8	-0.79	2.35	-6.35	4.78
Pre-treatment	1996	8	0.31	1.77	-3.89	4.50
Pre-treatment	1997	8	0.88	1.04	-1.59	3.35
Pre-treatment	1998	8	0.39	0.63	-1.10	1.89
Treatment	1999	8	-1.34	0.84	-3.33	0.65
Treatment	2000	8	-4.35	1.18	-7.15	-1.55

Treatment	2001	8	-8.15	1.67	-12.08	-4.21
Treatment	2002	8	-12.15	2.45	-17.94	-6.36
Treatment	2003	8	-15.90	3.51	-24.20	-7.60
Treatment	2004	8	-19.14	4.62	-30.07	-8.21
Treatment	2005	8	-21.39	5.63	-34.70	-8.08
Treatment	2006	8	-22.02	6.27	-36.84	-7.20
Treatment	2007	8	-22.30	6.69	-38.12	-6.48
Treatment	2008	8	-22.17	7.06	-38.87	-5.46
Treatment	2009	8	-21.33	7.13	-38.19	-4.47
Treatment	2010	8	-19.82	7.03	-36.44	-3.20
Treatment	2011	8	-18.17	6.70	-34.02	-2.32
Treatment	2012	8	-17.97	6.69	-33.79	-2.14
Treatment	2013	8	-17.80	6.70	-33.64	-1.96
Treatment	2014	8	-17.65	6.70	-33.49	-1.82
Treatment	2015	8	-17.73	6.65	-33.45	-2.01
Treatment	2016	8	-17.61	6.54	-33.08	-2.14
Treatment Period	1999-2016	18 yrs	-16.50	1.44	-19.55	-13.45

S2.8 Comparative funding between treatment and synthetic control countries during the treatment period (1999-2016)

In this section we checked our main analysis by observing per capita funding during the treatment period to identify whether differential funding patterns between treatment countries and synthetic control countries might explain the significant treatment effect observed in the main analysis, and as predicted by the theory of change. In this section we explored three indicators of funding: USAID funding for maternal and child health (MCH) and malaria[1]; (2) Net official development assistance (ODA) per capita and (3) Total health expenditures per capita[2].

Figure 2.8a below combines ODA per capita and USAID MCH/Malaria funding per capita to display the total average per capita funding separately by the eight synthetic control countries versus the eight treatment countries. The funding over the treatment period does not indicate any advantage of the treatment countries over the synthetic control countries: the synthetic control counties experience a higher level of per capita funding with an increasing trend over the treatment period; in contrast, in the treatment countries, the per capita funding decreases over the latter half of the treatment period. The differential funding pattern in this figure suggests that a change in total per capita funding in the treatment period, mainly from sources external to USAID, does not explain the treatment effect in the main analysis. (Note: a reminder that external funding was controlled for in the pre-treatment period using synthetic control procedures)

In Figure 2.8b below, ODA per capita and USAID MCH/Malaria funding per capita are shown separately to display the average per capita funding of each type separately by the eight synthetic control countries versus the eight treatment countries. ODA per capita follows the same pattern as in Figure 2.8a: the synthetic control counties experience a higher level of ODA per capita funding with an increasing trend over the treatment period; in contrast, in the treatment countries, the ODA per capita funding decreases over the latter half of the treatment period. Differential ODA per capita, between synthetic control countries and treatment countries does not explain the treatment effects in the main analysis.

USAID MCH/Malaria funding per capita is a small portion of total per capita funding in both synthetic control and treatment countries, with annual levels ranging from $0 per capita to $2.0 per capita. Among the eight synthetic control countries, only two countries received USAID MCH/Malaria funding during the treatment period: (1) Eritrea received six years of funding between 1999 and 2004, at an average that was less than $1 per capita; (2) Niger received one year of funding (2008) at a level of $0.03 per capita. In contrast, all eight treatment countries received continuous funding through the treatment period---although the funding amounts are not available for the years 2005 and 2006 and are show as missing from Figure 2.8b. The average per capita USAID MCH/Malaria spending increased in treatment countries from $0.2 per capita in 1999 to $1.7 per capita in 2016 with some in between years at higher levels (2010-2015).

This differential funding pattern is consistent with the main analysis and supports the theory of change (Figure 1) about expectations of USAID funding during periods of major health initiatives like IMCI. The treatment countries received significantly more funding and more consistent funding than the synthetic control countries. The treatment effect calculated in main analysis supports the hypothesis that countries with substantial levels of USAID funding during periods of major health initiatives like IMCI would see greater reductions in under-five mortality than countries with less USAID funding and less consistent funding. In addition, the USAID funding pattern shown in Figure S2.8b supports a dose-response relationship, often used to support inference of causality. Over the treatment period, average

[1] USAID maternal and child health (MCH) funding during the period 1999-2016 was sourced from USAID's annual Reports to Congress on the Child Survival and Health Programs Fund for 1999-2004 and later from the US State Department's Foreign Assistance Coordination and Tracking System financial reporting system from 2007-2016 (not available to the public). In the 1999-2004 period, funding of malaria programs was included in the Child Survival and Health Program funding and not available separately. From 2007-2016, MCH and malaria funds were separated and therefore, these two funds were combined to provide a consistent tracking of funds for the 1999-2016 period.

[2] Data on net ODA per capita and health expenditures per capita were downloaded from idea.usaid.gov on/about November 2017.

USAID funding across eight treatment countries showed an increasing trend, while USAID funding staggered and stopped in the synthetic control countries by 2009. In the main analysis, a similar increasing trend in the treatment effect over the treatment period was observed: in 1999 the treatment effect was -2.2 but by 2016 it was -38.5. This happened despite net ODA per capita increasing more in the control countries than in the treatment countries over the same period; the main advantage the treatment countries experienced was in USAID funding, although small portion of the total, a predicted by the theory of change.

As a last check, Figure S2.8c displays total average health expenditures per capita separately for synthetic control countries and treatment countries. As compared to total health expenditures per capita, USAID funding for MCH and Malaria is a very small proportion (see Figure S2.8b). A funding pattern inconsistent with the theory of change and the main analysis, would be one where total health expenditures (external and internal) in treatment countries, on a per capita basis, was greater than in the control countries during the treatment period (this was already controlled for in the pre-treatment period). However, in Figure 2.8c we observe the opposite. Per capita health expenditures are higher in the control countries. In addition, per capita health expenditures in the control countries increase sharply year over year, increasing the gap each year over the treatment countries.

In summary, external and/or internal funding (apart from USAID funding) does not appear to explain the treatment effects of the main analysis. The pattern of USAID funding, however, is consistent with the treatment effects in the main analysis and is consistent with the theory of change. USAID MCH and Malaria funding increased in treatment countries over time relative to control countries whereas the opposite was true of Net ODA or total health expenditures.

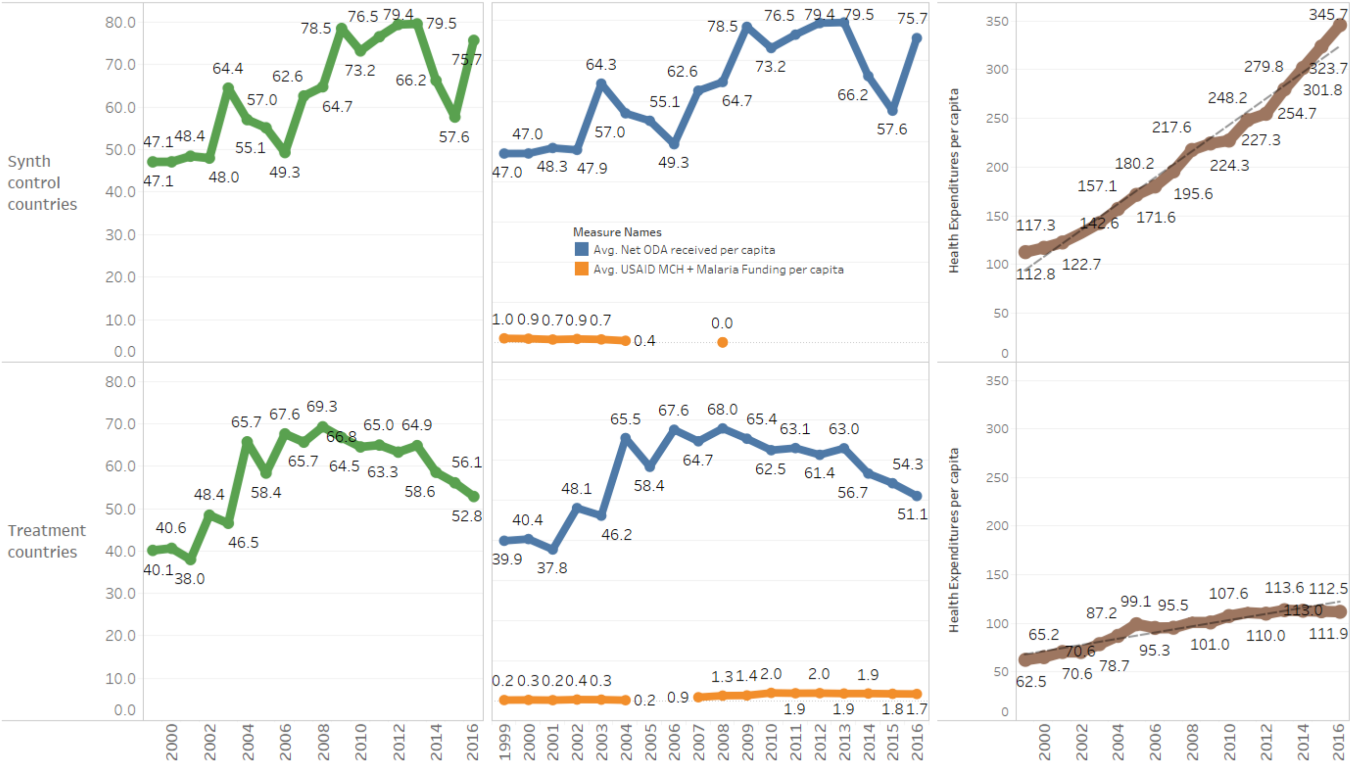

Figure S2.8a. Total Average Per capita funding (ODA + USAID MCH/Malaria), Treatment vs Synthetic Control Countries

Figure S2.8b. Average Per capita funding (USAID MCH Malaria vs Net ODA) - Treatment vs Synthetic Control Countries

Figure S2.8c. Average Total Health Expenditures Per capita - Treatment vs Synthetic Control Countries

S2.9 Restricting countries in the donor pool to those receiving fewer years of USAID funding

The donor pool in the main analysis is restricted to countries with less than 8 years of funding during the 18-year treatment period. This was done to prevent an overly small 'n' of countries leading to a potential 'beta error' of failing to statistically detect a true treatment effect. In this step, we check the results of carrying out the synthetic control method using more stringent restrictions: (1) countries with less than four years of USAID funding in the treatment period; and (2) countries with no USAID funding during the treatment period. In this check we find similar treatment effects as the main analysis. In retrospect this is not surprising as (mentioned above) as only two of the eight countries with positive weight that constitute the synthetic control received any USAID funding during the treatment period. Eritrea received funding during the first six years of the treatment period only. Niger received funds in only one year (2018). Further, these two countries constituted only a total of 11% to the weight of the synthetic control (Table 4).

Table S2.9a: Treatment effect estimates under two scenarios: (1) donor pool limited to countries with less than four years of USAID funding in the treatment period; and (2) donor pool limited to countries with no USAID funding during the treatment period. Effect size estimates are in units of U5MR. P-values are exact, empirical p-values based on placebo testing. Standardization involves dividing the effect size by RMSPE.

	**Less than four years of USAID Funding**			**No USAID Funding**		
**Year**	**Estimates**	**1-Sided p-values**	**Standardized 1-sided p-values**	**Estimates**	**1-Sided p-values**	**Standardized 1-sided p-values**
1999	-2.06	0.293	0.195	-1.72	0.382	0.206
2000	-5.46	0.171	0.000	-4.72	0.235	0.029
2001	-9.92	0.098	0.000	-8.94	0.176	0.001
2002	-14.79	0.049	0.024	-13.80	0.118	0.001
2003	-19.72	0.049	0.024	-18.85	0.029	0.001
2004	-24.28	0.049	0.024	-23.68	0.029	0.001
2005	-27.89	0.049	0.024	-27.62	0.029	0.001
2006	-30.03	0.073	0.024	-30.67	0.029	0.001
2007	-31.84	0.073	0.024	-33.10	0.029	0.001
2008	-33.31	0.073	0.024	-34.77	0.029	0.001
2009	-34.13	0.073	0.024	-36.05	0.029	0.001
2010	-34.25	0.073	0.000	-36.79	0.029	0.001
2011	-34.12	0.049	0.024	-37.81	0.001	0.001
2012	-35.32	0.024	0.024	-39.07	0.001	0.001
2013	-36.06	0.024	0.024	-39.94	0.001	0.001
2014	-36.59	0.024	0.024	-40.64	0.001	0.001
2015	-37.13	0.024	0.024	-41.23	0.001	0.001
2016	-37.11	0.024	0.024	-41.02	0.001	0.001
	Average pre-intervention RMSPE p-value: 0.46<br>Average treatment period standardized p-value: 0.024			Average pre-intervention RMSPE p-value: 0.53<br>Average treatment period standardized p-value: 0.001		

Donor pool limited to countries with less than four years of USAID funding in the treatment period

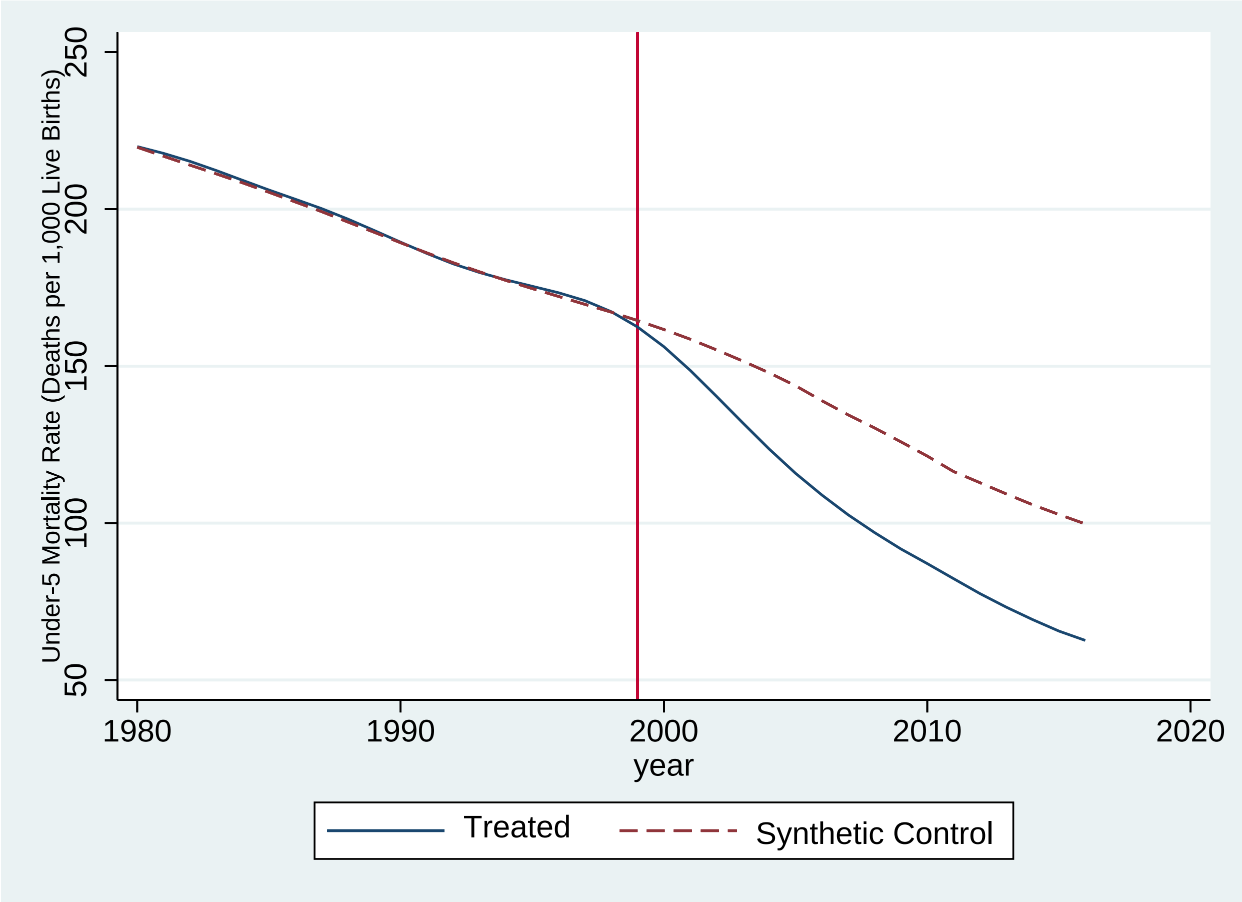

Figure S2.9a Synthetic control analysis using a donor pool limited to countries with less than four years of USAID funding in the treatment period

Table S2.9b: Predictor means between synthetic and test case in pre-intervention period, Donor pool with less than four years of funding

Variables	Real	Synthetic
TFR	6.56	6.72
Stunting	49.22	47.15
HIV	5.1	1.39
DPT	47.73	31.65
Sanitation	17.1	18.36
Clean water	40.3	43.89
Logged GDP	6.08	6.49
Urbanization	23.36	23.35
ODA per capita	41.06	41.39
Polity score	-2.9	-3.05
Under-5 mortality (1998)	167.31	167.18
Under-5 mortality (1990)	189.46	189.27
Under-5 mortality (1980)	219.89	219.67

Donor pool limited to countries with no USAID funding in the treatment period

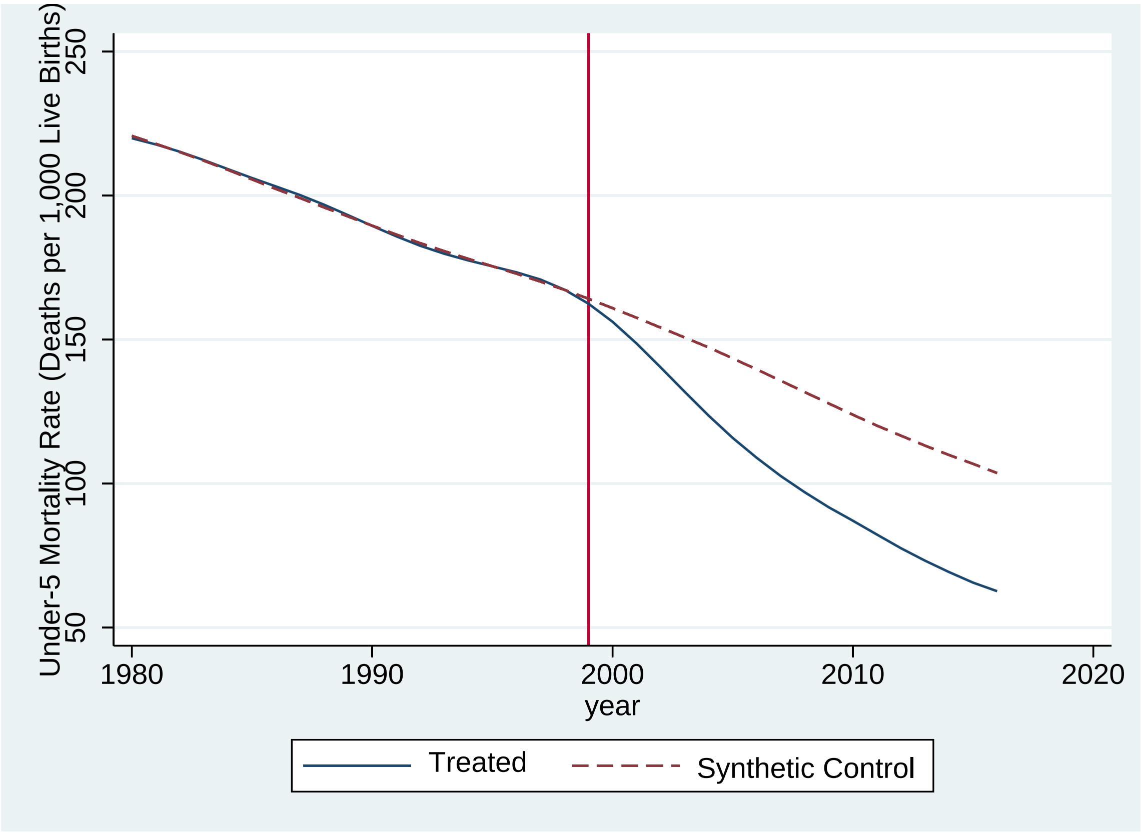

Figure S2.9b Synthetic control analysis using a donor pool limited to countries no USAID funding in the treatment period

Table S2.9c: Predictor means between synthetic and test case in pre-intervention period, Donor pool with no USAID funding

Variables	Real	Synthetic
TFR	6.56	6.7
Stunting	49.22	49.7
HIV	5.1	0.81
DPT	47.73	26.04
Sanitation	17.1	13.07
Clean water	40.3	42.26
Logged GDP	6.08	6.45
Urbanization	23.36	23.69
ODA per capita	41.06	41.09
Polity score	-2.9	-3.09
Under-5 mortality (1998)	167.31	167.3
Under-5 mortality (1990)	189.46	189.54
Under-5 mortality (1980)	219.89	220.71

S2.10. Repeated random assignment of eight countries in the donor pool into single control units for calculating alternative treatment effects and placebo testing

In the main analysis, the treatment unit is a grouped average of the eight Quadrant 1 treatment countries. The placebo test in the main analysis uses the 48 individual countries in the donor pool for placebo testing. In this sensitivity analysis, we created groups of eight countries each from those in the donor pool to act as placebos, matching the way that the eight treatment countries were grouped to create a single treatment unit in the main analysis. This was done to make the donor units used in the placebo testing more comparable to the grouped treatment unit. The 48 countries in the donor pool were randomly assigned to 48 grouped donor units of eight countries each.

The treatment effects of this sensitivity analysis were consistent with the main analysis, but with a lower treatment effect size (-19/1000 vs -29/1000). Standardized p-values from the grouped placebo testing were all significant (p. < .001).

Table S2.10a: Treatment effect estimates using grouped average donor units for placebo testing. Effect size estimates are in units of U5MR. P-values are exact, empirical p-values based on grouped unit placebo testing. Standardization involves dividing the effect size by RMSPE.

Year	Estimates	1-sided p-values	Standardized 1-sided p-values
1999	-5.10077	0.043	0.001
2000	-7.69243	0.001	0.001
2001	-11.0202	0.001	0.001
2002	-14.5229	0.001	0.001
2003	-17.8095	0.001	0.001
2004	-20.549	0.001	0.001
2005	-22.3745	0.001	0.001
2006	-23.3062	0.001	0.001
2007	-23.7609	0.001	0.001
2008	-23.7056	0.001	0.001
2009	-23.4804	0.001	0.001
2010	-22.9714	0.001	0.001
2011	-22.7332	0.001	0.001
2012	-22.8835	0.001	0.001
2013	-23.0715	0.001	0.001
2014	-23.2628	0.001	0.001
2015	-23.4704	0.001	0.001
2016	-23.3211	0.001	0.001

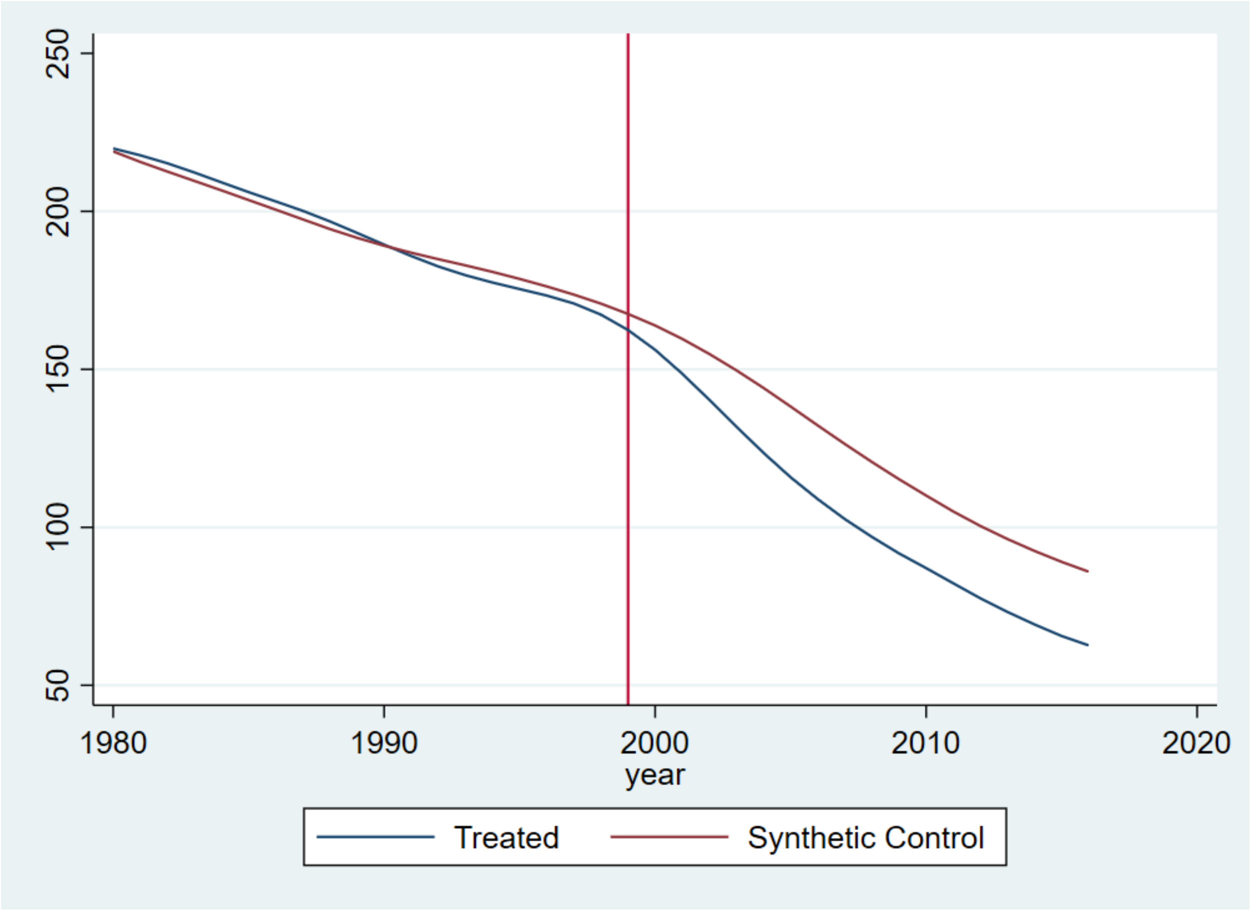

Figure S2.10a Synthetic control analysis using a donor pool of 48 groups of eight randomly selected countries

Table S2.10b Predictor Means for SCA with grouped donor pool units

Variable	Treated	Synthetic
TFR	6.56	6.75
Stunting	49.2	47.32
HIV	5.1	2.25
DPT	47.73	30.3
Sanitation	17.1	13.51
Clean Water	40.3	43.17
GDP (log)	6.08	6.22
Urbanization	23.36	22.15
ODA per capita	41.06	40.97
Polity Score	-2.9	-4.58
U5MR 1998	167.3	170.80
U5MR 1990	189.5	189.13
U5MR 1980	219.9	218.98

RMSPE : 2.516447

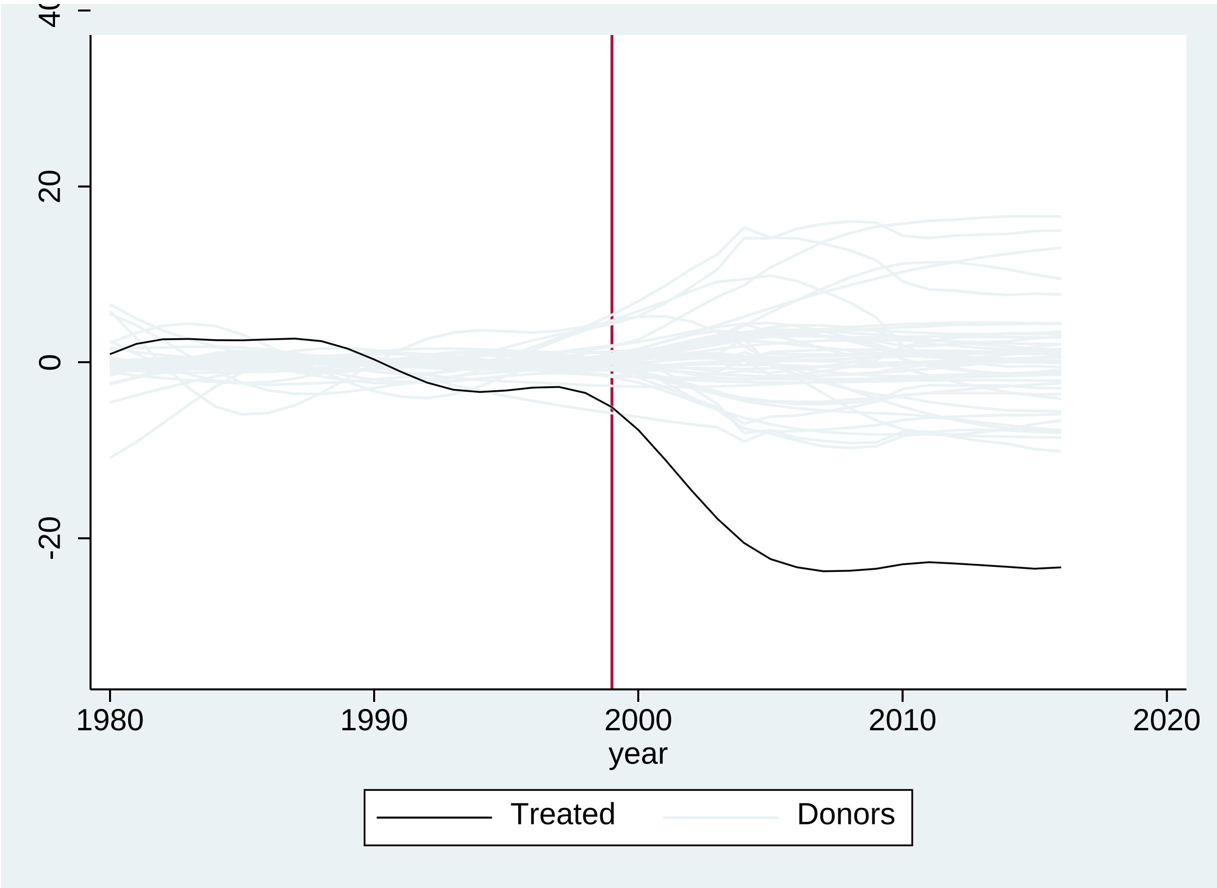

Figure S2.10b Placebo testing using a donor pool of 48 groups of eight randomly selected countries

S2.11. Results of a Difference-in-Difference analysis using the same donor pool and treatment units used in the main analysis

A longitudinal difference-in-difference analysis (similar to an analysis by an author's [Cohen]) was carried out using the same eight treatment countries and the same donor pool as the main analysis.[9] Because the outcome variable of interest (U5MR) is a rate, a random-intercept Poisson model was used to conduct the difference-in-difference analysis. Control variables were identical to the synthetic control analysis. Parallel assumptions for U5MR were plausible between treatment and donor countries.

The outcome of the model supports the main analysis (see Table S.11a). Results are reported as Incidence Rate Ratios. The three-way interaction term reveals the annual change in U5MR after the intervention period (1999) for treatment countries compared to donor countries. Being one of the eight treatment countries equals a decrease of 2.6% more in U5MR per year in the treatment period [(1 – 0.974)*100] as compared to a non-treatment country. This is consistent with the results of the main analysis where the treatment effect increases year-over-year during the treatment period.

Table S2.11.a: Treatment effects (incidence rate ratios) using a Difference-in-Difference analysis

Variables	Coefficient (IRR on U5MR)		Robust p-values
Constant (U5MR per 1,000 live births)	108.2	***	0.000
TFR	1.062		0.263
DPT3	0.998	*	0.0801
Sanitation access %	0.995		0.213
Clean water access %	1.001		0.730
Log(GDP per capita)	0.970		0.404
ODA per capita	1.000		0.596
Urbanization %	0.998		0.770
Polity IVR	1.002		0.640
Year	0.988	***	0.00359
Treatment country = 1	1.830	***	0.00357
Treatment period = 1	1.382	***	1.99e-05
Treatment country*Treatment period	1.541	***	0.000742
Treatment country*Treatment period*Year (DiD result)	0.974	***	0.000124
Observations	1,990		
Number of groups	56		

IRR = Incidence rate ratio
Robust p-values: ** p<0.01, ** p<0.05, * p<0.1
Treatment country = 1=yes or 0=No
Treatment period = before (0) or equal/after (1) 1999

References

1. Abadie A, Diamond A, Hainmueller J: **Synthetic Control Methods for Comparative Case Studies: Estimating the Effect of California's Tobacco Control Program**. Cambridge, Mass.: National Bureau of Economic Research; 2007.
2. Kreif N, Grieve R, Hangartner D, Turner AJ, Nikolova S, Sutton M: **Examination of the Synthetic Control Method for Evaluating Health Policies with Multiple Treated Units**. *Health Econ* 2016, **25**(12):1514-1528.
3. **STATALIST: The STATA Forum** [https://www.statalist.org/forums/forum/general-stata-discussion/general/1311745-different-ordering-of-predictors-yields-different-answers-using-synth-in-stata]
4. Abadie A, Diamond A, Hainmueller J: **Synthetic Control Methods for Comparative Case Studies: Estimating the Effect of California's Tobacco Control Program**. *Journal of the American Statistical Association* 2010, **105**(490):493-505.
5. **Synthetic Control Method App** [https://wishes.shinyapps.io/intervention_effect_study/]
6. Carling KL, Y.: **The Power of the Synthetic Control Method.** . In: *Working Papers in Transport, Tourism, Information Technology and Microdata Analysis.* 2016.
7. Rudholm N, Y L, Carling K: **How Does Big-Box Entry Affect Labor Productivity in Durable Goods Retailing? A Synthetic Control Approach.** . In: *HUI Working Paper* Stockholm, Sweden; 2018.
8. Lepine A, Lagarde M, Le Nestour A: **Free primary care in Zambia: an impact evaluation using a pooled synthetic control method**. In. Health, Econometrics and Data Group (HEDG) Working Papers 15/20: HEDG, c/o Department of Economics, University of York; 2015.
9. Cohen RL, Li Y, Giese R, Mancuso JD: **An evaluation of the President's Emergency Plan for AIDS Relief effect on health systems strengthening in sub-Saharan Africa**. *J Acquir Immune Defic Syndr* 2013, **62**(4):471-479.
